# Impaired hydrogen sulfide biosynthesis underlies eccentric contraction–induced force loss in dystrophin-deficient skeletal muscle

**DOI:** 10.1172/JCI176942

**Published:** 2025-01-14

**Authors:** W. Michael Southern, Erynn E. Johnson, Elizabeth K. Fasbender, Katherine S. Fallon, Courtney L. Cavazos, Dawn A. Lowe, George G. Rodney, James M. Ervasti

**Affiliations:** 1 Department of Biochemistry, Molecular Biology, and Biophysics, University of Minnesota, Minneapolis, Minnesota, USA.; 2 Department of Integrative Physiology, Baylor College of Medicine, Houston, Texas, USA.; 3 Department of Family Medicine and Community Health, Division of Physical Therapy and Rehabilitation Science, University of Minnesota, Minneapolis, Minnesota, USA.

**Keywords:** Metabolism, Muscle biology, Neuromuscular disease, Proteomics, Skeletal muscle

## Abstract

Eccentric contraction–induced (ECC-induced) force loss is a hallmark of murine dystrophin-deficient (*mdx*) skeletal muscle that is used to assess efficacy of potential therapies for Duchenne muscular dystrophy. While virtually all key proteins involved in muscle contraction have been implicated in ECC force loss, a unifying mechanism that orchestrates force loss across such diverse molecular targets has not been identified. We showed that correcting defective hydrogen sulfide (H_2_S) signaling in *mdx* muscle prevented ECC force loss. We also showed that the cysteine proteome of skeletal muscle functioned as a redox buffer in WT and *mdx* muscle during ECCs, but that buffer capacity in *mdx* muscle was significantly compromised by elevated basal protein oxidation. Finally, chemo-proteomic data suggested that H_2_S protected several proteins central to muscle contraction against irreversible oxidation through persulfidation-based priming. Our results support a unifying, redox-based mechanism of ECC force loss in *mdx* muscle.

## Abstract 2

Treating dystrophin-deficient skeletal muscle with hydrogen sulfide fully protects against eccentric contraction-induced force loss.

## Graphical abstract

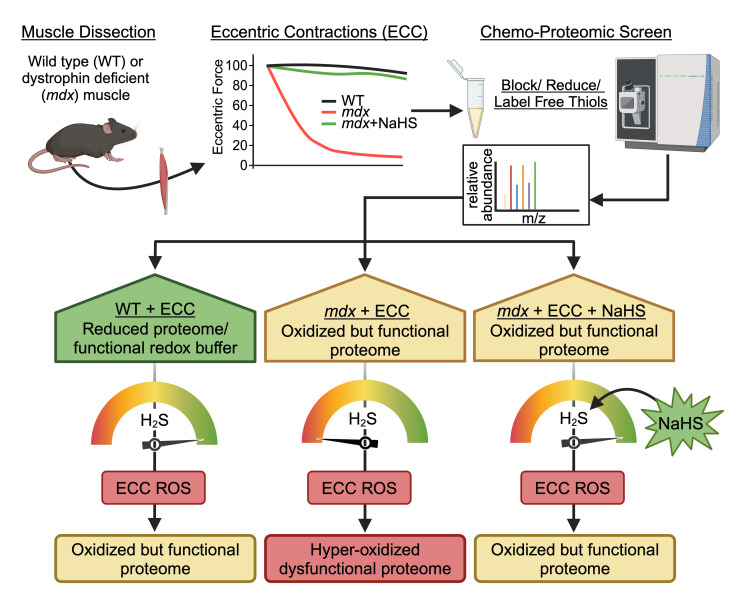

## Introduction

The most widely studied murine model of Duchenne muscular dystrophy (DMD) is the *Dmd^mdx^* mutant mouse (*mdx*) (1, 2), which is characterized by the absence of the protein dystrophin with consequent severe muscle pathology including membrane damage, Ca^2+^ dysregulation, elevated reactive oxygen species (ROS), and aberrant cell signaling (3). Beginning with the landmark in vitro studies by Moens et al. (4) and Petrof et al. (5), rapid eccentric contraction–induced (ECC-induced) force loss has proven to be one of the most robust and reproducible phenotypes of *mdx* skeletal muscle. ECC force loss has been an important metric to gauge therapeutic efficacy of various potential experimental drugs or gene therapies for the treatment of DMD, but the mechanism of this acute force loss appears complex and remains poorly understood.

Since the ECC force loss phenotype was first documented, multiple groups have demonstrated the phenomenon across a range of *mdx* skeletal muscles using a variety of methods (Supplemental Table 1; supplemental material available online with this article; https://doi.org/10.1172/JCI176942DS1). Collectively, these reports have provided clues into the possible mechanisms of force loss by either directly studying the mechanism or testing the efficacy of potential therapeutic interventions. Independent of utrophin upregulation, studies have implicated membrane excitability, calcium signaling, myofibrillar dysfunction, and redox homeostasis as mediators of ECC force loss in *mdx* muscle (Supplemental Table 1). However, it is not clear whether and how such a diverse array of molecular targets may be coordinately regulated to effect rapid and dramatic force drop.

Redox signaling could be a unifying mechanism linking together the various factors of *mdx* ECC force loss, as we and others have shown that force loss can be partially prevented by manipulation of cellular redox status (6–8). For example, *N*-acetylcysteine (NAC) partially protects *mdx* muscles against ECC force loss (6, 8), which could be due to its antioxidant function (9). However, ROS scavenging by NAC is limited because of the slow reaction kinetics relative to endogenous cellular antioxidants like superoxide dismutase, catalase, and glutathione (10). NAC has also been shown to act through the transsulfuration pathway (TSP), which is responsible for the metabolism of cysteine into precursors for glutathione (GSH), taurine, and hydrogen sulfide (H_2_S) (10). H_2_S is a gasotransmitter that can regulate skeletal muscle redox balance and muscle signaling through protein thiol persulfidation–mediated protection from irreversible oxidative thiol modifications (i.e., sulfinylation, sulfonylation) (11). Several studies have reported TSP disruptions in dystrophin-deficient muscle (12–15), which provides a plausible link between the TSP and NAC-derived protection from ECC force loss.

Here we present evidence suggesting that the protective effects of NAC on *mdx* skeletal muscle were mediated through the TSP. We demonstrated that aberrant TSP metabolism in *mdx* muscle was restored to WT by expression of a full-length dystrophin/utrophin chimera, and that H_2_S supplementation prevented ECC force loss in *mdx* extensor digitorum longus (EDL) muscles. Physiological and chemo-proteomic data showed that the cysteine proteome redox buffer in *mdx* muscle was compromised, leaving proteins involved in muscle contraction or its regulation susceptible to hyperoxidation, which was prevented by H_2_S supplementation via protein persulfidation. Together, our data demonstrated that high basal levels of protein oxidation, aberrant TSP metabolism, and excess ROS generated during ECCs combine to produce ECC force drop in *mdx* skeletal muscle.

## Results

### NAC-dependent protection of mdx muscle against ECC force loss is mimicked by exogenous l-cysteine.

**Figure 1:**
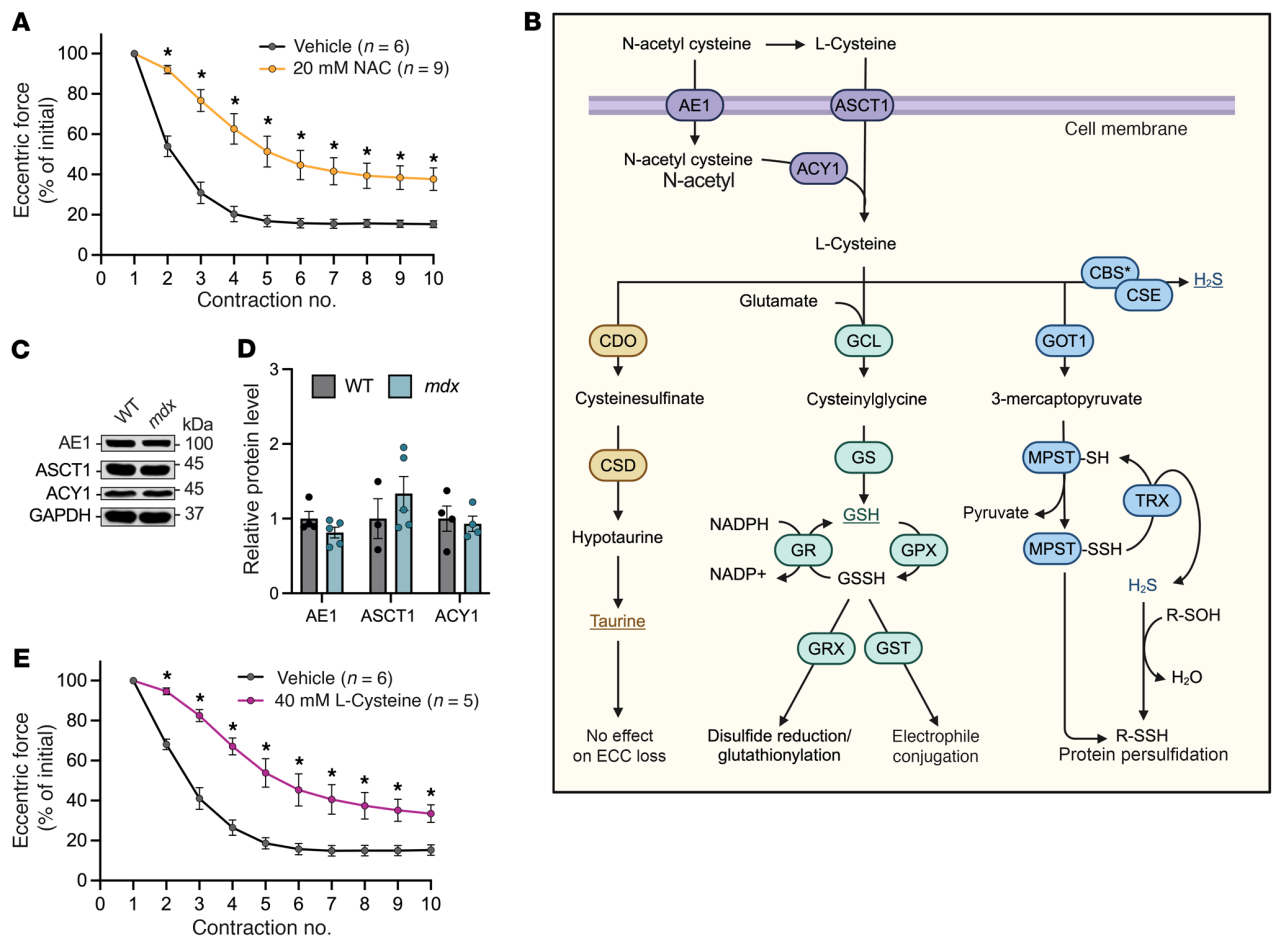
NAC-dependent protection of *mdx* muscle against ECC force loss is mimicked by exogenous l-cysteine. (**A**) Change in eccentric force during 10 ECCs in *mdx* EDL muscles incubated with vehicle or 20 mM *N*-acetylcysteine (NAC). (**B**) Putative mechanisms of NAC cell entry and contribution to cysteine metabolism through the 3 arms of the transsulfuration pathway. (**C** and **D**) Representative results (**C**) and quantification (**D**) from immunoblot analysis of NAC/cysteine membrane transport proteins from WT and *mdx* gastrocnemius muscles. (**E**) Change in eccentric force during 10 ECCs in *mdx* EDL muscles incubated with vehicle or 40 mM l-cysteine. All ECC force data in **A** and **E** are expressed as a percentage of the force generated during the first ECC. Results are presented as mean ± SEM. *P < 0.05 by 2-way repeated-measures ANOVA in **A** and **E**.

NAC partially protects *mdx* EDL muscles against ECC force loss (Figure 1A), but the mechanism by which it protects *mdx* skeletal muscle from ECC is unknown. NAC reportedly acts through the TSP (10), which is the singular metabolic pathway for cysteine metabolism in mammals (16). With l-cysteine as a substrate, the TSP can be largely divided into 3 arms: taurine biosynthesis, GSH biosynthesis, and H_2_S biosynthesis (Figure 1B). NAC can be either deacetylated and then imported into the cell via cysteine importers (i.e., ASCT1) or directly imported into the cell via anion exchanger 1 (AE1), where it is subsequently deacetylated by aminoacylase-1 (ACY1) (10). Western blot analysis confirmed that AE1, ASCT1, and ACY1 were all similarly expressed in WT and *mdx* skeletal muscle (Figure 1, C and D), which supported the idea that NAC treatment led to an increase in free l-cysteine. Indeed, l-cysteine treatment prevented ECC force loss in *mdx* skeletal muscle (Figure 1E) to the same extent as observed with NAC (Figure 1A).

While *mdx* skeletal muscle taurine levels are reduced compared with WT (17), others have previously shown that increasing intramuscular taurine levels in *mdx* mice did not prevent ECC force loss in EDL muscles (18). Thus, we focused subsequent study on the GSH and H_2_S biosynthesis arms of the TSP.

### NAC treatment of mdx muscle restores reduced GSH to WT levels.

**Figure 2:**
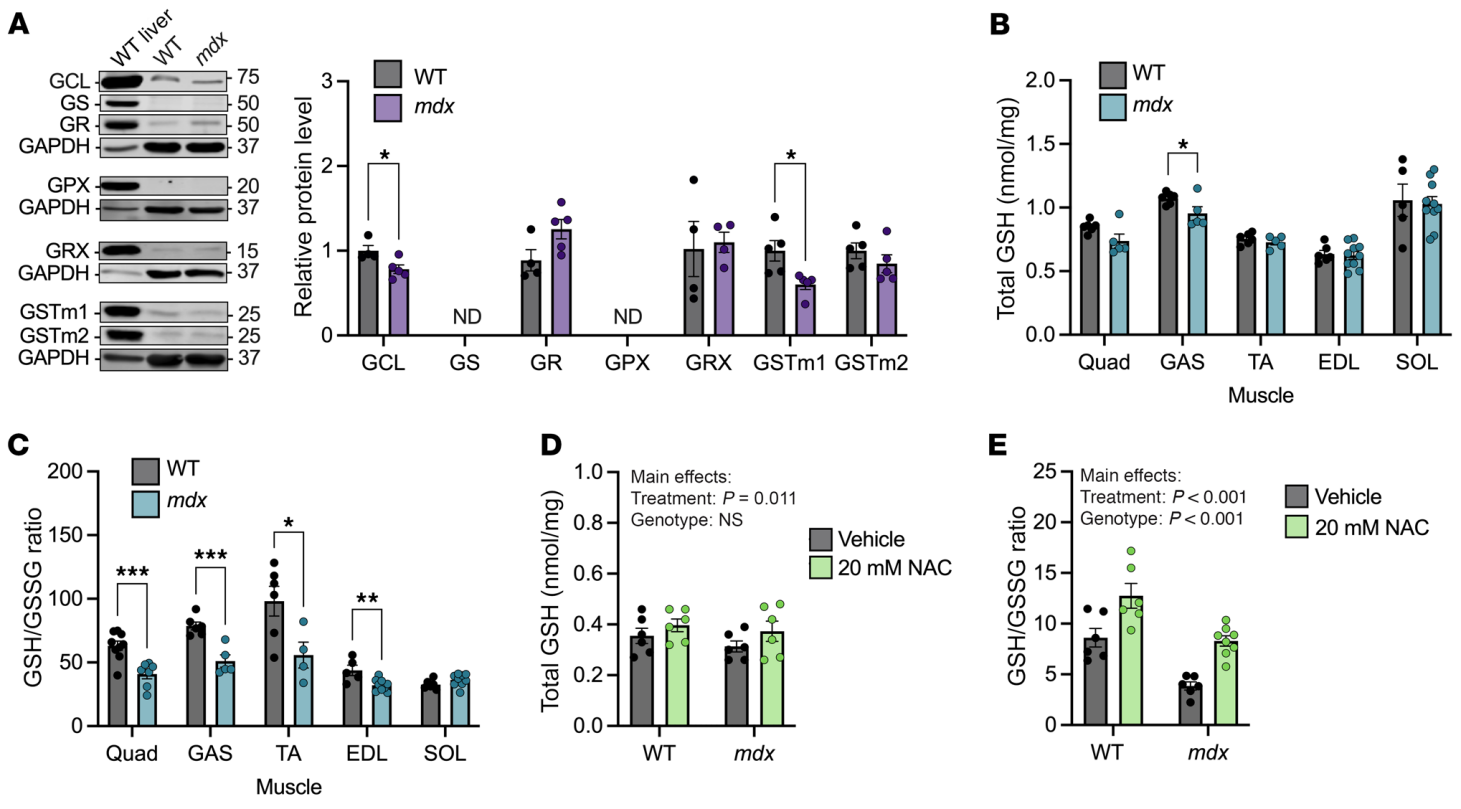
NAC treatment of *mdx* muscle restores reduced GSH to WT levels. (**A**) Representative immunoblot results and quantification of GSH synthesis and recycling protein levels from WT and *mdx* gastrocnemius (GAS) muscles. Immunoblot samples run contemporaneously have been grouped and presented with a representative loading control image. (**B**) Levels of total GSH in quadriceps (Quad), GAS, tibialis anterior (TA), EDL, and soleus (SOL) muscles from WT and *mdx* mice. (**C**) Ratio of reduced to oxidized GSH in Quad, GAS, TA, EDL, and SOL muscles from WT and *mdx* mice. (**D**) Levels of total GSH in WT and *mdx* EDLs incubated with vehicle or 20 mM NAC for 1 hour. (**E**) Ratio of reduced to oxidized GSH in WT and *mdx* EDLs incubated with vehicle or 20 mM NAC for 1 hour. Results are presented as mean ± SEM. *P < 0.05, **P < 0.01, ***P < 0.001 by Student’s *t* test in **A**–**C**, or main effects by 2-way ANOVA reported in **D** and **E**.

To assess whether NAC acted through the GSH arm of the TSP to effect protection from ECC force drop, we performed Western blot analysis of WT and *mdx* skeletal muscle to probe for differences in enzymatic regulators of GSH synthesis and downstream GSH signaling. We found small but significant deficits in glutamate-cysteine ligase (GCL) and glutathione *S*-transferase mu-1 (GSTm1) in *mdx* muscle compared with WT (Figure 2A). However, even though GCL is a key rate-limiting enzyme of GSH synthesis, a deficit in GCL was not accompanied by a loss in total GSH measured across different *mdx* muscles, except for a small reduction in *mdx* gastrocnemius muscle (Figure 2B). Despite near-normal total GSH in *mdx* muscles, we found that with the exception of the soleus muscle, the ratio of reduced GSH to oxidized GSH (GSH/GSSG) was significantly reduced across all *mdx* muscles compared with WT (Figure 2C), which is consistent with previous studies and indicative of an elevated oxidative stress (19, 20).

We next tested whether NAC could alter the GSH/GSSG ratio by incubating WT and *mdx* EDL muscles in vitro in the absence or presence of NAC. NAC treatment elevated total GSH as well as the GSH/GSSG ratio in WT and *mdx* EDL muscles (Figure 2, D and E). These data showed that NAC treatment could restore the GSH/GSSG ratio in *mdx* muscle to WT levels, possibly through de novo GSH synthesis.

### TSP enzyme levels are diminished in mdx skeletal muscle.

**Figure 3:**
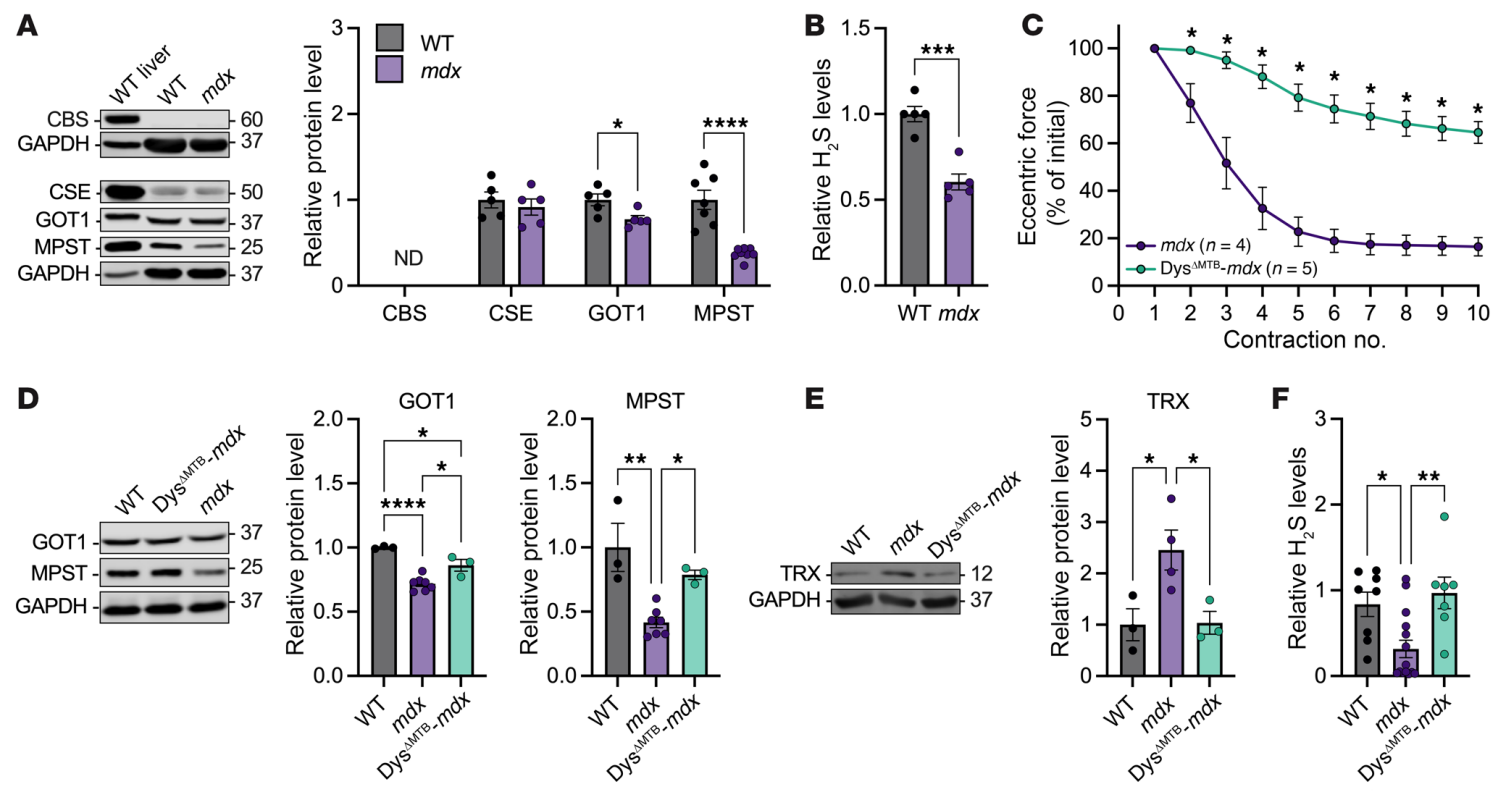
Diminished TSP enzyme levels in *mdx* skeletal muscle. (**A**) Representative immunoblot results and quantification of H_2_S-generating enzyme protein levels from WT and *mdx* gastrocnemius (GAS) muscles. Immunoblot samples run contemporaneously have been grouped and presented with a representative loading control image. (**B**) Relative levels of H_2_S in GAS muscle of WT and *mdx* mice. (**C**) Change in eccentric force during 10 ECCs in Dys^ΔMTB^-*mdx* and littermate *mdx* EDL muscles; eccentric forces are expressed as a percentage of the force generated during the first ECC. (**D** and **E**) Representative immunoblot results and quantification of GOT1, MPST, and TRX protein levels from WT, *mdx*, and Dys^ΔMTB^-*mdx* GAS muscles. (**F**) Relative levels of H_2_S in GAS muscle of WT, *mdx*, and Dys^ΔMTB^-*mdx*. Results are presented as mean ± SEM. *P < 0.05, ***P < 0.001, ****P < 0.0001 by Student’s *t* test in **A** and **B**; *P < 0.05, **P < 0.01, ****P < 0.0001 by 1-way ANOVA in **D**–**F**; *P < 0.05 by 2-way repeated-measures ANOVA in **C**.

To investigate the H_2_S synthesis arm of the TSP, we Western-blotted WT and *mdx* muscle for enzymes involved in H_2_S synthesis (Figure 1B). Cystathionine β-synthase (CBS) and cystathionine γ-lyase (CSE) are 2 enzymes responsible for generation of H_2_S via cellular metabolism of homocysteine to l-cysteine. Consistent with publicly available gene expression data for mouse skeletal muscle (MuscleDB; ref. 21), CBS was not detected in either WT or *mdx* skeletal muscle. CSE was detected but was not different between WT and *mdx* gastrocnemius tissue (Figure 3A). Alternatively, H_2_S can also be produced via conversion of l-cysteine to 3-mercaptopyruvate by glutamic-oxaloacetic transaminase 1 (GOT1) and mercaptopyruvate sulfurtransferase (MPST). H_2_S is generated during the sulfur transfer from MPST to a dithiol acceptor such as thioredoxin (TRX). Both GOT1 and MPST were significantly reduced in *mdx* skeletal muscle compared with WT (Figure 3A). In contrast, TRX was significantly elevated in *mdx* skeletal muscle compared with WT (Figure 3E). Consistent with decreased GOT1 and MPST abundance, H_2_S levels were significantly decreased in *mdx* muscle compared with WT (Figure 3B). As we have previously shown (22), *mdx* mice transgenically expressing a full-length dystrophin/utrophin chimera (Dys^ΔMTB^-*mdx*) are rescued of all dystrophic phenotypes, including ECC force loss (Figure 3C). Here, we found that GOT1, MPST, and TRX proteins were all restored to WT levels in transgenic Dys^ΔMTB^-*mdx* muscle (Figure 3, D and E), as were H_2_S levels (Figure 3F). These data demonstrated a link between skeletal muscle dystrophin expression and H_2_S synthesis that correlated with protection against ECC force loss in *mdx* muscle.

### Sodium hydrogen sulfide supplementation prevents ECC force loss in mdx EDLs.

**Figure 4:**
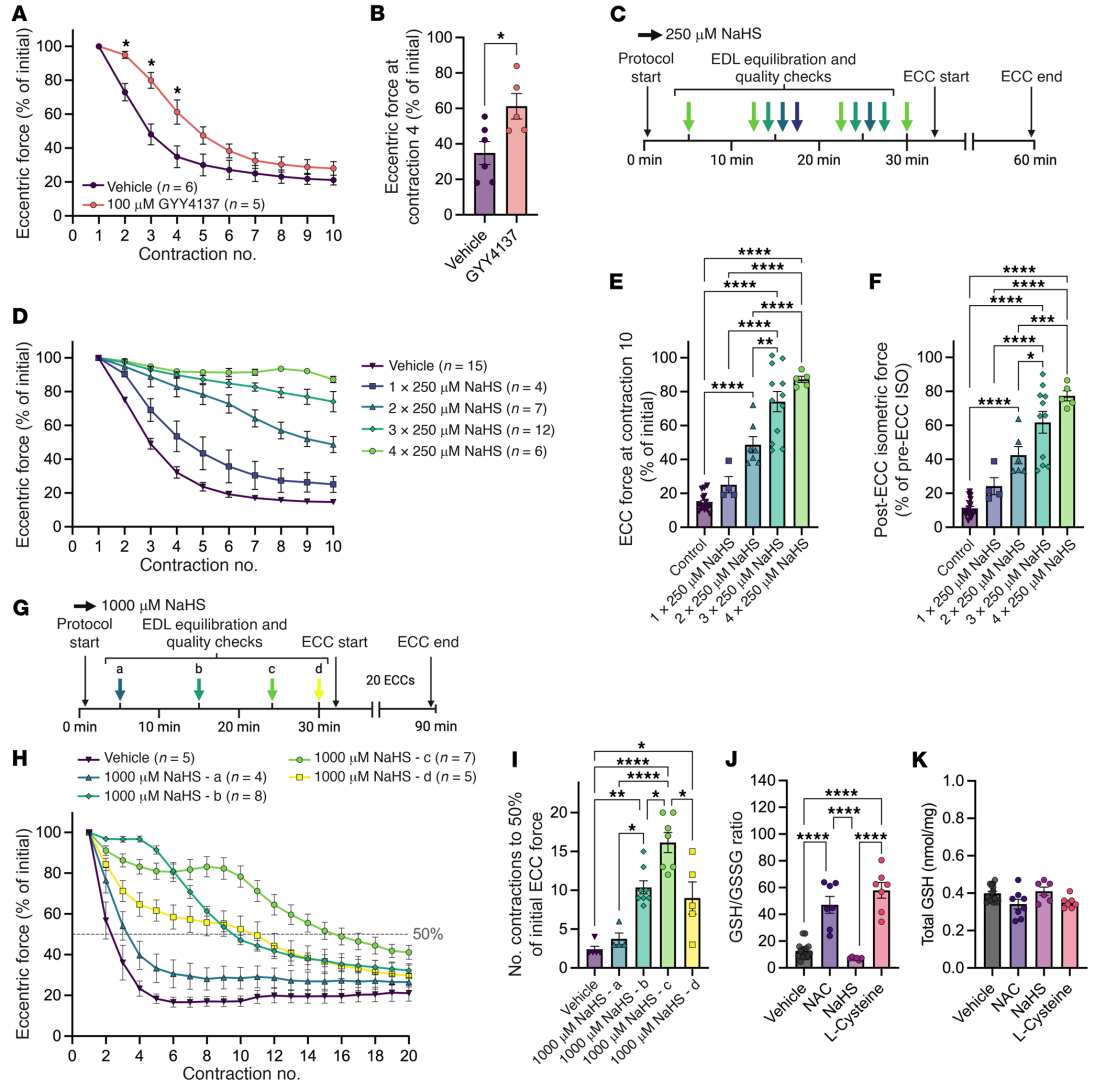
NaHS supplementation prevents ECC force loss in *mdx* EDLs. (**A**) Change in eccentric force during 10 ECCs in *mdx* EDL muscles incubated with vehicle or 100 μM GYY4137. (**B**) Eccentric force produced at ECC #4 in **A**. (**C**) Illustration of dosing protocols used to test the effects of repeated additions of NaHS on ECC force loss. (**D**) Change in eccentric force during 10 ECCs in *mdx* EDL muscles incubated with vehicle or 1, 2, 3, or 4 doses of 250 μM NaHS. (**E**) Eccentric force produced at ECC #10 in **D**. (**F**) Post-ECC isometric (ISO) force from *mdx* EDLs subjected to 10 ECCs and various concentrations of NaHS in **D**. (**G**) Schematic illustrating the protocols used to test the temporal effects of a single 1,000 μM NaHS dose on ECC force loss. (**H**) Change in eccentric force during 20 ECCs in *mdx* EDL muscles incubated with vehicle or 1,000 μM NaHS at minute 2, 15, 25, or 30. (**I**) Number of ECCs to reach 50% of initial ECC force in **H**. (**J** and **K**) Ratio of reduced to oxidized GSH (**J**) and levels of total GSH (**K**) in *mdx* EDLs incubated with vehicle, 20 mM NAC, 750 μM NaHS, or 40 mM l-cysteine for 1 hour. All ECC force data in **A**, **B**, **D**, **E**, and **H** are expressed as a percentage of the force generated during the first ECC. Results are presented as mean ± SEM. *P < 0.05 by Student’s *t* test in **B**; *P < 0.05, **P < 0.01, ***P < 0.001, ****P < 0.0001 by 1-way ANOVA in **E**, **F**, and **I**–**K**; *P < 0.05 by 2-way repeated-measures ANOVA in **A**.

Based on the data described thus far, we hypothesized that NAC-mediated protection against ECC force drop in *mdx* EDLs was due to elevated l-cysteine metabolism via GOT1 and MPST, presumably resulting in an acute increase in H_2_S production. We further hypothesized that NAC may only partially protect against ECC force drop owing to limited H_2_S production resulting from decreased levels of GOT1 and MPST enzymes in *mdx* muscle. To test this hypothesis, we treated *mdx* EDLs with the water-soluble, slow-releasing H_2_S donor GYY4137, which bypasses GOT1 and MPST to directly act on downstream H_2_S targets (23). In agreement with our hypothesis, GYY4137 protected against ECC force drop, but only partially, which suggested that GYY4137 does not sufficiently elevate H_2_S levels (Figure 4, A and B). Therefore, we treated *mdx* EDLs with the rapid-releasing H_2_S donor sodium hydrogen sulfide (NaHS), which hydrolyzes immediately in water to generate large, transient spikes in H_2_S levels (23, 24). We tested the effect of varying amounts of NaHS by repeated delivery of 250 μM NaHS doses into the in vitro bath throughout the protocol (Figure 4C). The justification for repeated additions of NaHS was 2-fold: (a) to systematically increase the extra- and intramuscular concentration of H_2_S and (b) to counter gaseous diffusion out of the bath and maintain elevated H_2_S levels throughout the duration of the protocol. A strong dose-dependent response was observed, with 4 doses of NaHS amounting to complete protection against ECC force loss in *mdx* EDLs (Figure 4, D–F), indicating that H_2_S deficiency drives ECC force drop in *mdx* muscle.

Additionally, data in Figure 4D suggested that NaHS operated through a protective priming of the muscle that was dependent on the duration of exposure to NaHS. Thus, to investigate the extent to which the protective effect of NaHS treatment was transient, *mdx* muscles were treated with NaHS and subjected to 20 ECCs. In this experiment we treated *mdx* EDLs with a large dose of NaHS (1,000 μM) at 4 different time points in the protocol to characterize the optimal treatment timing (Figure 4G). Irrespective of when NaHS was administered, all treatment groups eventually lost ECC force, suggesting that the protective effects of NaHS were transient (Figure 4H). Further, the time point at which NaHS was administered determined the level of protection, suggesting that timing of NaHS treatment relative to the first ECC was necessary for maximal protection (Figure 4I).

Because both NAC and NaHS could protect against ECC force loss, and NAC restored the GSH/GSSG ratio in *mdx* EDLs to WT levels (Figure 2E), we hypothesized that NaHS would also restore the GSH/GSSG ratio. Therefore, we treated *mdx* EDLs with NAC, NaHS, and l-cysteine (as a positive NAC-like control; Figure 1D) for 1 hour in the in vitro bath and measured both total GSH and the GSH/GSSG ratio. Surprisingly, while l-cysteine treatment mirrored the increase in the GSH/GSSG ratio produced by NAC treatment, NaHS had no effect on the ratio, indicating that NaHS treatment operated independently of GSH and that protection from ECC force loss was not dependent on reduced GSH (Figure 4, J and K).

### Reactive protein targets of H_2_S are elevated in mdx skeletal muscle.

**Figure 5:**
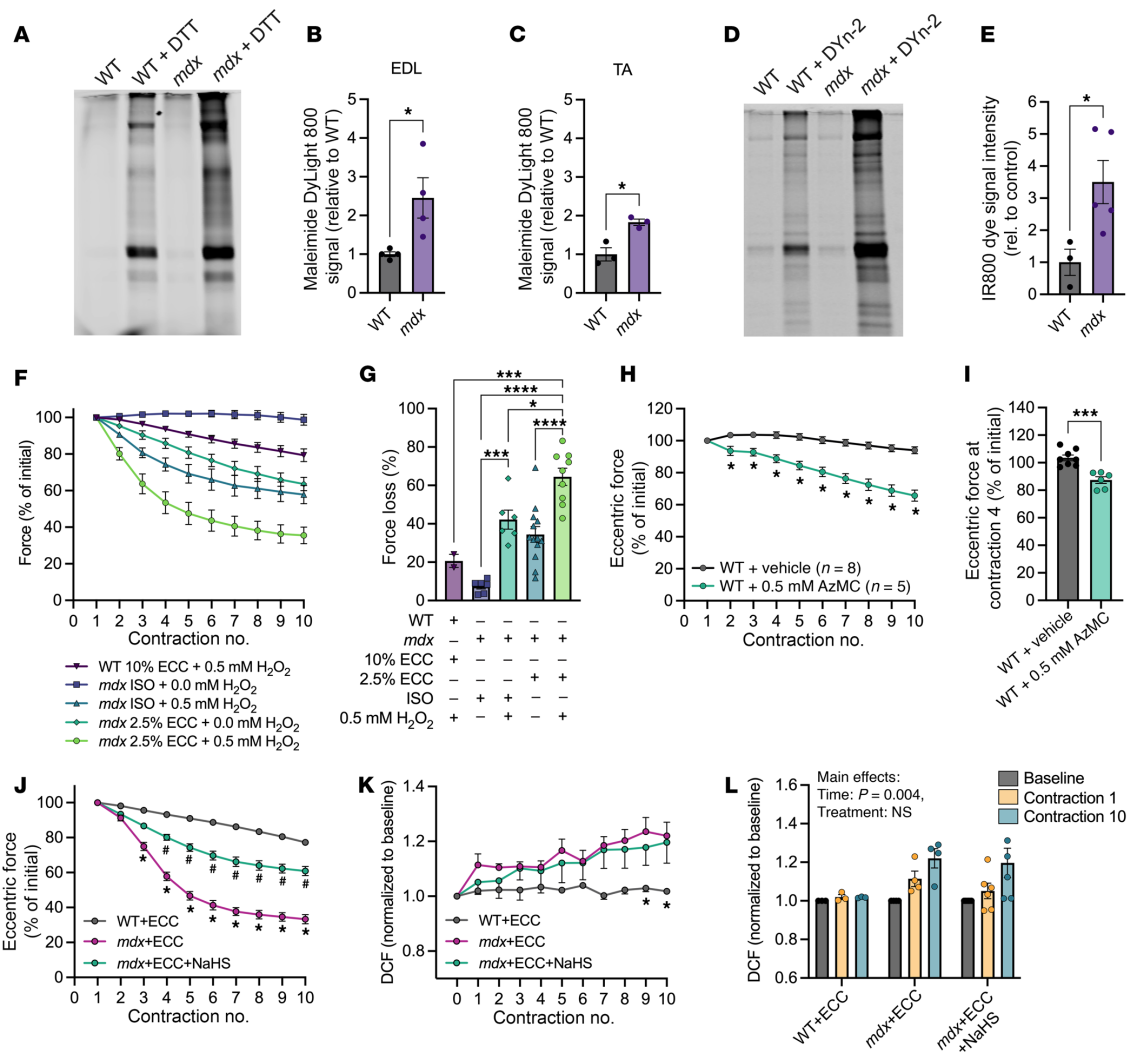
Reactive protein targets of H_2_S are elevated in *mdx* skeletal muscle. (**A**–**C**) Representative image (**A**) and quantification of in-gel fluorescence showing protein thiol oxidation in WT and *mdx* EDL (**B**) or tibialis anterior (TA) (**C**) muscles. (**D** and **E**) Representative image (**D**) and quantification of in-gel fluorescence showing protein thiol sulfenylation from WT and *mdx* gastrocnemius muscle (**E**). (**F**) Change in force during 10 isometric (ISO) or eccentric contractions in WT or *mdx* EDLs incubated with or without hydrogen peroxide (H_2_O_2_). (**G**) Percentage force loss after 10 ISOs or ECCs from the groups presented in **F**. (**H**) Change in eccentric force during 10 ECCs in WT EDL muscles treated with vehicle or 0.5 mM of the H_2_S scavenger 7-azido-4-methylcoumarin (AzMC). (**I**) Eccentric force produced at ECC #4 in **H**. (**J**) Change in eccentric force during 10 ECCs in *mdx* EDL muscles incubated with vehicle or NaHS. (**K**) Change in DCF fluorescence, a proxy for ROS production, from pre-ECC baseline through 10 ECCs in the EDL muscles from **J**. (**L**) DCF fluorescence during baseline, after ECC #1, and after ECC #10 in **K**. All ISO or ECC force data in **F**, **H**, and **J** are expressed as a percentage of the force generated during the first contraction. Results are presented as mean ± SEM. *P < 0.05, ***P < 0.001 by Student’s *t* test in **B**, **C**, **E**, and **I**; *P < 0.05, ***P < 0.001, ****P < 0.0001 by 1-way ANOVA in **G**; *P < 0.05 by 2-way repeated-measures ANOVA in **H**. *Different from WT+ECC and *mdx*+ECC+NaHS, ^#^different from WT+ECC and *mdx*+ECC, *P* < 0.05 by 2-way repeated-measures ANOVA in **J**. *Different from *mdx*+ECC, *P* < 0.05 by 2-way repeated-measures ANOVA in **K**. Main effects by 2-way ANOVA reported in **J**.

H_2_S does not directly interact with reduced protein thiols (–SH), but rather with reversibly oxidized thiol derivatives such as cysteine sulfenic acid (–SOH), cysteine disulfides (–SSR), glutathiolated cysteine (–SSG), and *S*-nitrosylated cysteine (–SNO) to produce cysteine persulfides (–SSH) (11, 25, 26). Therefore, we assessed whether *mdx* muscle has more H_2_S-reactive oxidized protein thiols than WT muscle using fluorescently labeled maleimide. Total cysteine oxidation was higher in the *mdx* EDL and tibialis anterior muscles compared with WT (Figure 5, A–C), suggesting that *mdx* muscles have elevated reactive protein targets for H_2_S. Labeling with a sulfenic acid–specific probe, Dyn-2 (27), revealed significantly greater cysteine sulfenylation in *mdx* muscle compared with WT (Figure 5, D and E), which was notable because sulfenylation is the oxidized thiol derivative that is most reactive with H_2_S (25). Elevated levels of baseline sulfenylation in *mdx* muscle, combined with excess ECC ROS from sources such as Nox2, predicted impaired protein function due to an increased susceptibility of protein thiols to irreversible hyperoxidation (i.e., sulfinylation [–SO_2_H] and sulfonylation [–SO_3_H]). To test whether *mdx* muscle was more sensitive than WT to increased ROS during ECCs as previously suggested (28), we treated WT and *mdx* EDL muscles with hydrogen peroxide (H_2_O_2_) to simulate an increase in excess ROS. To avoid masking the effects of excess H_2_O_2_ due to rapid force loss in *mdx* EDLs during ECCs, we adjusted our standard ECC protocol from a 10% length change to the less stressful 2.5% length change, which does not result in rapid force loss (29). We further tested whether performing non-lengthening isometric (ISO) contractions in the presence of exogenous H_2_O_2_ would produce force loss similar to that of lengthening contractions. The addition of exogenous H_2_O_2_ led to force drop during ISO contractions (Figure 5, F and G; blue triangles vs. purple squares). Even greater force loss was observed when 2.5% ECCs were combined with H_2_O_2_, which was suggestive of an additive effect of ECC and exogenous ROS (Figure 5, F and G; green circles vs. teal diamonds). In stark contrast, WT EDL muscles showed minimal drop even when challenged with the same H_2_O_2_ concentration and the standard 10% length change (Figure 5, F and G; purple inverted triangles). Interestingly, scavenging endogenous H_2_S with an excess of 7-azido-4-methylcoumarin (30, 31) was sufficient to cause a significant approximately 35% force loss in WT muscles challenged with 10% ECC (Figure 5, H and I) that was similar in magnitude to that observed in *mdx* EDLs challenged with 2.5% ECC (Figure 5, F and G), but less than half the force loss in *mdx* EDL muscles challenged with 10% ECC (Figure 1, A and E, Figure 3C, and Figure 4, A and D). Consistent with our and others’ previous studies (6, 7), *mdx* mice ablated for Nox2 activity (*mdx*/p47^–/–^) were partially protected from ECC force loss (Supplemental Figure 1, A and B), as were *mdx* mice treated with 2 different Nox2 inhibitors, gp91ds-TAT (32) and GSK2795039 (33) (Supplemental Figure 1, C–F). Together, these data suggested that elevated baseline protein thiol oxidation and decreased H_2_S levels made *mdx* skeletal muscle more susceptible than WT to irreversible thiol oxidation from excess ROS produced by sources such as Nox2 during ECCs.

### NaHS acts on targets of ROS and not the source(s).

Because ROS has only been previously measured as a function of passive stretch in *mdx* muscle fibers (6, 7, 34), we adapted our horizontal in vitro physiology/imaging apparatus to measure ROS via dichlorofluorescein (DCF) fluorescence as a function of ECC. As in the classic vertical in vitro system used to measure ECC force loss in Figure 1 and Figures 3–5, *mdx* EDLs underwent significant force loss compared with WT EDLs when subjected to ECCs in the horizontal system, which could be attenuated with NaHS treatment (Figure 5J). As indicated by increasing DCF fluorescence, ROS levels increased over the course of 10 ECCs in both *mdx*+ECC and *mdx*+ECC+NaHS EDLs relative to WT. However, neither the rate nor the final amount of DCF fluorescence was different between the *mdx* treatment conditions (Figure 5, K and L). These data indicate that H_2_S did not protect *mdx* EDLs from force loss by blocking the sources of ECC ROS, but rather H_2_S protected the protein thiol targets of ECC ROS.

### The cysteine proteome of skeletal muscle functions as a ROS buffer during ECCs.

**Figure 6:**
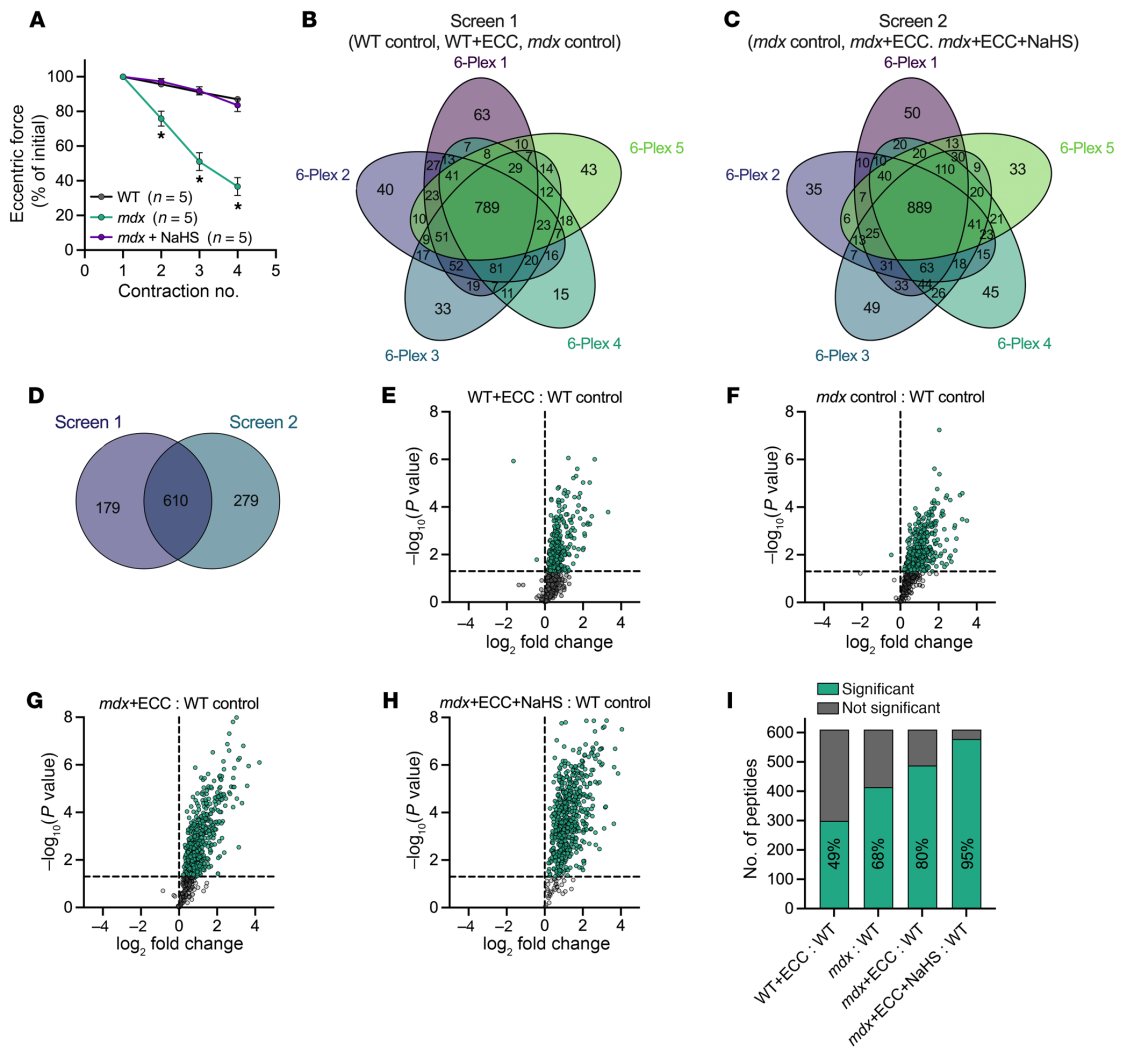
The cysteine proteome of skeletal muscle functions as a ROS buffer during ECCs. (**A**) Change in eccentric force during 4 ECCs in WT and *mdx* EDL muscles. Data are expressed as a percentage of the force generated during the first ECC. Contralateral EDLs were also isolated and incubated without ECCs or NaHS for control conditions. (**B** and **C**) Venn diagrams showing the peptide sequence overlap across five 6-plexes within screen 1 (**B**) or screen 2 (**C**). Plex 1 (of 5) within screen 1 contained 1 (of 5) biological replicate EDL from WT control, WT+ECC, and *mdx* control conditions, while plex 1 (of 5) in screen 2 contained 1 (of 5) biological EDL replicate from *mdx* control, *mdx*+ECC, and *mdx*+ECC+NaHS conditions. (**D**) Venn diagram showing peptide sequence overlap between the 789 peptides from screen 1 and 889 peptides from screen 2. The 610 common peptides were used for subsequent analysis. (**E**–**H**) Volcano plots showing the oxidation level relative to the WT control group of individual peptides from the WT+ECC (**E**), *mdx* control (**F**), *mdx*+ECC (**G**), and *mdx*+ECC+NaHS (**H**) groups. Green data points above the horizontal dashed line indicate *P* < 0.05 versus the WT control group. (**I**) Percentage of 610 peptides from WT+ECC, *mdx* control, *mdx*+ECC, and *mdx*+ECC+NaHS groups with significantly (green bars) different levels of oxidation versus WT control group. Percentages derived from significant peptides in **E**–**H**. Results are presented as mean ± SEM. *P < 0.05 by 2-way repeated-measures ANOVA in **A**.

To better understand how NaHS treatment prevents ECC force drop in *mdx* skeletal muscle (Figure 4D), we employed iodoTMT chemo-proteomics, which allowed for identification and relative quantitation of reversibly oxidized cysteine residues of proteins in WT and *mdx* muscle as a function of ECC and NaHS treatment. Paired 6-plex proteomic screens were performed with biological EDL replicates isolated from WT and *mdx* mice to evaluate differences in thiol oxidation levels across all groups (Supplemental Figure 2A). Screen 1 contained WT (*n* = 5 EDLs), WT+ECC (*n* = 5 EDLs), and *mdx* control (*n* = 5 EDLs) groups, while screen 2 contained the *mdx* control (for inter-screen normalization) (*n* = 5 EDLs), *mdx*+ECC (*n* = 5 EDLs), and *mdx*+ECC+NaHS (*n* = 5 EDLs) groups. For WT ECC, *mdx*+ECC, and *mdx*+ECC+NaHS conditions, EDL muscles were isolated from WT and *mdx* mice and subjected to 4 ECCs with or without NaHS treatment. For WT and *mdx* control conditions, contralateral EDLs were isolated and incubated in the in vitro bath for the same duration of time but without exposure to ECCs. Only 4 ECCs were performed in order to capture the state of protein oxidation at a point when *mdx* EDLs had achieved near-maximal force loss while the transient protective effect of NaHS was also maximal (Figure 4H). WT+ECC– and *mdx*+ECC+NaHS–treated EDLs lost only 13% and 16.5% force, respectively, over 4 contractions, while *mdx*+ECC EDLs lost 63.3% force (Figure 6A).

Using custom analysis routines in R, we identified overlapping peptide sequences across the five 6-plexes within each screen. Proteomic analysis was only performed on peptides that were commonly identified across all (5 of 5) 6-plexes for both screen 1 and screen 2. Within screen 1 we identified 789 common peptides, and in screen 2 we identified 889 common peptides (Figure 6, B and C). We combined and normalized the 2 screens using common *mdx* control samples within each screen and found 610 common peptides that were common across all plexes and screens. These peptides were used for all further analysis (Figure 6D). Peptide oxidation level for each muscle condition was expressed relative to the WT control group (Figure 6, E–I). These group comparisons against the WT control condition revealed 4 key findings: (a) the WT muscle proteome was oxidized following ECCs (Figure 6, E and I), (b) the *mdx* muscle proteome was oxidized at baseline (Figure 6, F and I), (c) ECCs also resulted in proteome oxidation in *mdx* muscle (Figure 6, G and I), and (d) ECCs and NaHS treatment resulted in even higher proteome oxidation in *mdx* muscle (Figure 6, H and I).

### The ROS buffer in mdx muscle is impaired during ECCs.

**Figure 7:**
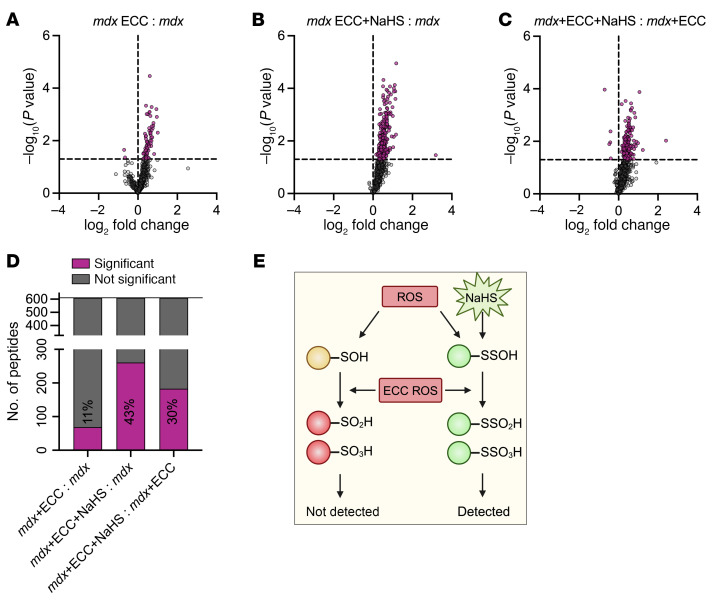
The ROS buffer in *mdx* muscle is impaired during ECCs. (**A**–**C**) Volcano plots showing the oxidation level relative to the *mdx* control group (**A** and **B**) or *mdx*+ECC (**C**) of individual peptides from the *mdx*+ECC (**A**), *mdx*+ECC+NaHS (**B**), and *mdx*+ECC+NaHS (**C**) groups. Purple data points above the horizontal dashed line indicate *P* < 0.05 versus *mdx* control (**A** and **B**) or *mdx*+ECC (**C**) group. (**D**) Percentage of 610 peptides from *mdx*+ECC and *mdx*+ECC+NaHS groups with significantly (purple bars) different levels of oxidation versus *mdx* control or *mdx*+ECC groups. Percentages derived from significant peptides in **A**–**C**. (**E**) Proposed model explaining the greater number of significantly oxidized peptides in the *mdx*+ECC+NaHS group compared with the *mdx*+ECC group.

When expressed relative to the *mdx* control group, only 11% of peptides in the *mdx*+ECC group had significantly different oxidation levels compared with 43% of the peptides in the *mdx*+ECC+NaHS group (Figure 7, A, B, and D). When *mdx*+ECC+NaHS was compared with *mdx*+ECC, the NaHS-treated EDLs had 30% more significantly oxidized peptides (Figure 7, C and D). Together, these data indicated that NaHS treatment paradoxically resulted in additional protein thiol oxidation during ECCs. Because H_2_S only reacts with existing oxidized thiol derivatives, we proposed that this paradoxical result could be explained by irreversible protein thiol hyperoxidation in *mdx* muscle challenged by ECC that was prevented by H_2_S-mediated protective persulfidation (–SSH). The iodoTMT tags used in this study only detect reversibly oxidized protein thiols (Supplemental Figure 2, A and B). Thus, any irreversibly oxidized thiols produced in the *mdx*+ECC group would not be detected by our proteomic screens (Figure 7E). However, with NaHS treatment, reversibly oxidized protein thiols most likely underwent protective persulfidation modifications that prevented ECC-induced irreversible oxidation and enabled proteomic detection via iodoTMT tag labeling (Figure 7E).

### Enrichment analysis identified multiple proteins involved in muscle contraction and redox homeostasis.

**Figure 8:**
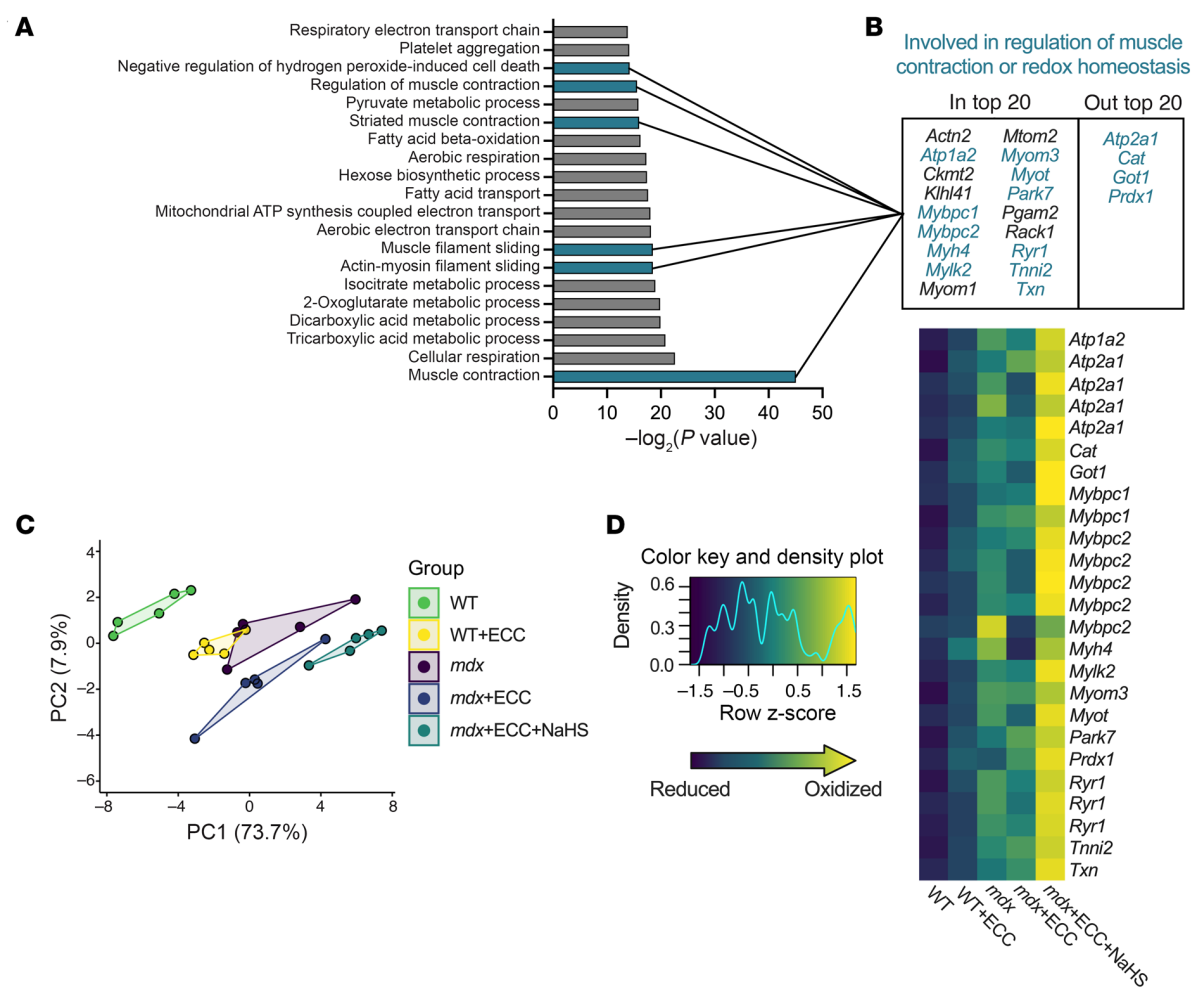
Enrichment analysis identifies multiple proteins involved in muscle contraction and redox homeostasis. (**A**) The top 20 enriched Biological Process GO terms from enrichment analysis performed on the 119 unique genes identified in Figure 7C. Blue bars signify GO terms related to muscle contraction or redox homeostasis. (**B**) List of unique genes identified from the muscle contraction or redox homeostasis GO terms highlighted in **A**. Four additional muscle contraction– or redox-related genes not represented in the top 20 GO terms were also included. (**C**) Principal component analysis (PCA) of average oxidation level between all samples colored by experimental group. Each sample is composed of the 24 peptides represented by the 15 unique genes highlighted in **B**. (**D**) Heatmap of *z* scores showing relative differences in cysteine oxidation level of individual peptides of interest between WT, WT+ECC, *mdx*, *mdx*+ECC, and *mdx*+ECC+NaHS groups.

Given the unique protein thiol oxidation signature in the *mdx*+ECC+NaHS group compared with *mdx*+ECC, we narrowed the focus of our analysis to the 119 unique proteins represented among the 185 significant peptides identified in this comparison (Figure 7C). Using the associated gene symbols for 119 unique proteins, we performed enrichment analysis using Enrichr (35–37). Figure 8, A and B, shows that the top 20 most enriched Biological Process Gene Ontology (GO) terms included several muscle- and redox-related terms: muscle contraction (GO:0006936; –log_2_ *P* value = 45.1), actin-myosin filament sliding (GO:0033275; –log_2_ *P* value = 18.5), muscle filament sliding (GO:0030049; –log_2_ *P* value = 18.5), striated muscle contraction (GO:0006941; –log_2_ *P* value = 16.0), and negative regulation of H_2_O_2_-induced cell death (GO:1903206; –log_2_ *P* value = 14.2). From these muscle- and redox-related GO terms, we identified 18 genes of interest, 11 of which were known to be directly related to muscle contraction and/or redox homeostasis (Figure 8B). Additionally, while not included in any of the top 20 GO terms, *Atp2a1* (SERCA), *Cat* (catalase), *Got1* (glutamic-oxaloacetic transaminase 1), and *Prdx1* (peroxiredoxin 1) were included in our analysis given their relevance to muscle contraction and redox homeostasis (Figure 8B and Supplemental Table 2). Principal component analysis of the 24 peptides in Supplemental Table 2, representing our 15 proteins of interest, revealed distinct clustering of all experimental groups from WT control and clear separation between the *mdx*+ECC and *mdx*+ECC+NaHS groups (Figure 8C). A heatmap was generated to examine individual peptide-level differences in oxidation between groups (Figure 8D). Overall, the *mdx*+ECC group had minimal differences compared with baseline *mdx* oxidation level, while peptides in the *mdx*+ECC+NaHS group had the highest oxidation level. These data suggested that cysteine residues in proteins important for contraction and redox homeostasis in *mdx* muscle were irreversibly oxidized during ECCs, which could be prevented by pretreatment with NaHS.

### NaHS treatment prevents irreversible oxidation of cysteine residues on several proteins implicated in mdx ECC force loss.

**Figure 9:**
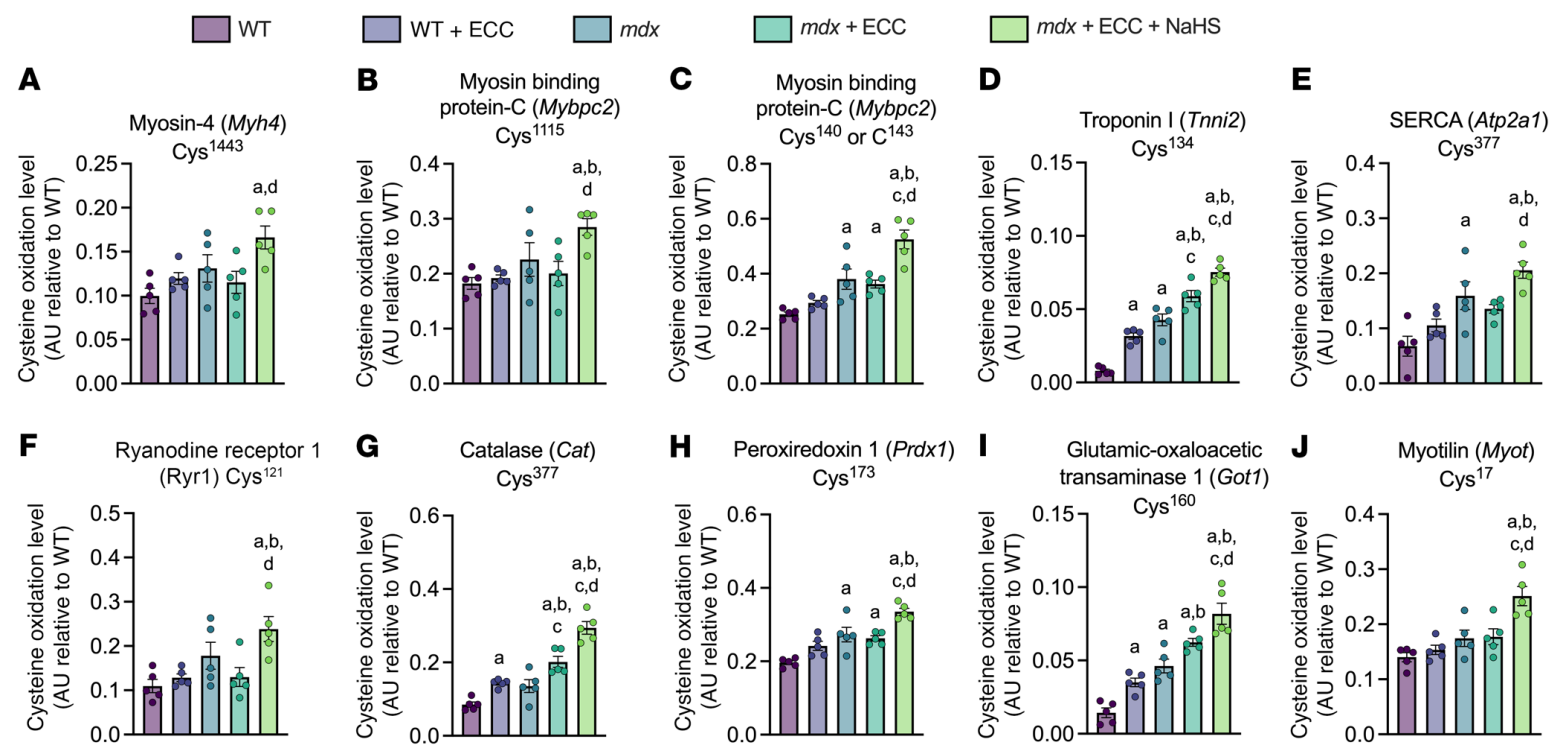
NaHS treatment prevents irreversible oxidation of cysteine residues on several proteins implicated in *mdx* ECC force loss. (**A**–**J**) Bar graphs of the average level of cysteine oxidation between WT, WT+ECC, *mdx*, *mdx*+ECC, and *mdx*+ECC+NaHS groups in arbitrary units (AU) expressed relative to WT. Protein cysteine residues that had significant pairwise differences between *mdx*+ECC and *mdx*+ECC+NaHS after 1-way ANOVA analysis are presented. Elevated cysteine residue oxidation in the *mdx*+ECC+NaHS condition indicates an NaHS-mediated abatement of suppressive irreversible oxidation following ECCs in the *mdx*+ECC condition. Results are presented as mean ± SEM. ^a^*P* < 0.05 vs. WT, ^b^*P* < 0.05 vs. WT+ECC, ^c^*P* < 0.05 vs. *mdx*, ^d^*P* < 0.05 vs. *mdx*+ECC by 1-way ANOVA in **A**–**J**.

To assess differences more rigorously in cysteine residue oxidation between the experimental groups, we performed an ANOVA across the 5 groups for each peptide/residue. We have presented the 10 cysteine residues and their respective proteins that remained significant (*P* ≤ 0.044) for pairwise differences in oxidation between *mdx*+ECC and *mdx*+ECC+NaHS groups (Figure 9, A–J). These data supported the idea that the function of several muscle contractile proteins previously implicated in ECC force loss in *mdx* skeletal muscle (Supplemental Table 1) was inhibited by irreversible hyperoxidation, which could be prevented by H_2_S-mediated persulfidation.

## Discussion

Rapid force loss during a series of ECCs is a hallmark of dystrophin-deficient skeletal muscle pathology. Many elements of muscle contraction contribute to force loss (Supplemental Table 1), but a unifying mechanism linking these elements together has yet to be identified. Here, we demonstrated that pharmacological supplementation of H_2_S fully protected *mdx* EDL muscles from ECC force loss (Figure 4), while H_2_S depletion caused ECC force loss in WT muscle (Figure 5, H and I). Chemo-proteomic data indicated that *mdx* muscle was susceptible to irreversible protein thiol hyperoxidation, and that H_2_S preserved ECC force by protectively priming key cysteine residues in a persulfidation-dependent manner (Supplemental Figure 3, A–C). Furthermore, we showed that while WT muscle also underwent proteome-wide oxidation during ECCs, low levels of baseline protein oxidation together with normal H_2_S production prevented irreversible thiol oxidation of key proteins that regulate or affect force production, and consequently, ECC force loss. Based on these data, we have presented a unifying redox-based mechanism of *mdx* ECC force loss in skeletal muscle that is dependent on 3 factors: (a) elevated baseline protein oxidation, (b) increased ROS generated during ECCs, and (c) an impaired H_2_S biosynthesis pathway (Supplemental Figure 3, A–C).

Our initial objective was to elucidate the mechanism by which NAC partially prevents ECC force loss in *mdx* muscle. NAC is typically thought to act via 3 distinct mechanisms that collectively provide antioxidant effects: reduction of disulfides, direct ROS scavenging, and serving as a precursor to the antioxidant GSH (9). One report highlights a fourth mechanism whereby NAC-derived free cysteine is channeled through the TSP to yield H_2_S (10). Our current data have led us to conclude that NAC partially protects *mdx* muscle from ECC force loss by partially restoring H_2_S levels caused by impaired TSP metabolism.

Our data showed that the TSP was impaired in 12-week-old *mdx* muscle, resulting in a deficit in H_2_S, and are in general agreement with additional studies. Panza et al. show lower mRNA levels for key enzymes in the TSP in both human DMD patients and *mdx* mice, while Ellwood et al. show lower CSE and MPST protein abundance in dystrophin/utrophin double-knockout mouse muscle (DKO), but not *mdx* muscle (12, 14). Relative to WT muscle, Panza et al. find lower H_2_S levels in old (17 weeks) but not young (7 weeks) *mdx* mice (14), while Ellwood et al. find lower H_2_S in DKO muscle (12) and dystrophin-deficient *Caenorhabditis* *elegans* muscle (13), but not muscle from 9-week-old *mdx* animals (12). Collectively, these data suggest a relatively steep, age-dependent decline in H_2_S that coincides with the absence of ECC force loss in young *mdx* mice (38).

We envision 2 primary mechanisms for the protective effects of NaHS treatment against ECC force loss in *mdx* EDLs. First, NaHS treatment directly induced persulfidation-dependent changes in several contraction-related proteins that collectively protected *mdx* muscle from force loss. This potential mechanism is supported by several reports that demonstrate that persulfidation directly modulates the function of GAPDH (39), K_v_7 channels (40), K_ATP_ channels (41), and muscle ring finger 1 (42). Alternatively, a second, more indirect mechanism of NaHS-mediated protection could have been a protective priming of the proteome via persulfidation that prevented irreversible hyperoxidation and loss of contractile protein function (43). While we do not have conclusive evidence for one mechanism over the other, we propose that our observations were more consistent with the protective priming mechanism of H_2_S action, for 3 reasons: (a) protection by NaHS displayed a post-treatment lag and was transient (Figure 4H); (b) NaHS did not reverse existing ECC force loss, but rather prevented further force loss (Figure 4H); (c) our chemo-proteomics data showed that peptides in the *mdx*+ECC+NaHS group had significantly higher reversible cysteine oxidation levels in comparison with the *mdx*+ECC group. This was an unexpected observation that was likely the result of greater irreversible thiol oxidation in peptides from the *mdx*+ECC group. H_2_S-mediated persulfidation protects proteins from hyperoxidation as increased oxidative stress in the absence of normal cellular H_2_S levels results in lower persulfidation, elevated sulfenylation, and irreversible sulfinylation and sulfonylation (43, 44). Our data support the hypothesis that H_2_S, in a persulfidation-dependent manner, indirectly maintained protein function through protection of susceptible thiols from attacks by excess ROS.

In agreement with multiple other studies (17, 34, 45–49), we have shown that baseline protein thiol oxidation was significantly elevated in *mdx* muscle compared with WT (Figure 2E, Figure 5, A–E, and Figure 6F). More interesting and unique to this study, our chemo-proteomic data also showed greater proteome oxidation after ECCs in both WT and *mdx* muscle, which indicated that ROS were generated during ECCs and readily react with protein thiols. Reactions between excess ROS and protein thiols are consistent with the known role of the proteome thiol pool serving as a ROS buffer and an important cellular defense mechanism during times of increased oxidative stress (50). WT muscle could withstand the oxidant insult since the proteome was in a reduced state and the muscle had a high ROS-buffering capacity. However, due to the elevated baseline oxidation, *mdx* muscle had a diminished buffering capacity, which rendered the muscle more susceptible than WT to irreversible oxidation and protein dysfunction, culminating in ECC force loss.

We also designed and employed an in vitro physiology system that enabled measurement of ROS levels during ECCs in an intact EDL muscle. We observed rising levels of ROS in *mdx* muscles over the course of 10 ECCs (Figure 5, K and L), which was consistent with our chemo-proteomic data that showed elevated cysteine oxidation levels after ECC, supporting long-held assumptions that ROS are generated during ECCs (6, 8). While protecting *mdx* muscle against ECC force loss, NaHS did not alter the rate of ROS generation (Figure 5, K and L), which indicates that NaHS does not affect sources of ECC ROS. Together, these data further reinforce that excess ECC ROS is one of 3 unique primary drivers of force loss in *mdx* muscle, and that the most effective means of protection against ECC force loss is realized at the targets of ROS, i.e., the thiol proteome, rather than the sources of ROS, i.e., NOX2 (6, 7).

Perturbations of distinct regulatory elements of muscle contraction in *mdx* skeletal muscle, such as calcium signaling and redox homeostasis, can elicit a common beneficial outcome, namely protection against ECC force loss (Supplemental Table 1). This implies a unifying mechanism of ECC force loss that operates across multiple force-generating pathways. Here, we proposed that oxidative stress is a likely candidate for such a mechanism as it is known to negatively impact an array of pathways involved in muscle contraction. In line with this notion, we presented evidence that H_2_S supplementation could afford broad persulfidation-dependent protection from irreversible protein thiol oxidation across contractile and redox proteomes that collectively preserved force production after ECC. Our data further suggested that elevated baseline thiol oxidation, excess ROS production during ECCs, and an impaired TSP rendered *mdx* proteins susceptible to function-compromising irreversible oxidation (Supplemental Figure 3, A–C). This paradigm can explain why several distinct proteins involved in muscle contraction, or its regulation, are implicated in ECC force loss, but only show partial protection when targeted individually.

As noted above, multiple components of the muscle contractile apparatus are implicated in *mdx* ECC force loss, with ROS potentially acting as the unifying mechanism of dysfunction across those components (51, 52). Consistent with a redox-mediated, pleiotropic mechanism of force loss, we identified 10 cysteine residues in 9 proteins that functionally span multiple levels of muscle contraction and redox homeostasis (Figure 9, A–J). The oxidation profile of these cysteine residues across the 5 experimental groups indicated that these residues are points of H_2_S-mediated protection against irreversible forms of thiol oxidation and could be key contributors to ECC force loss. With regard to Ca^2+^ signaling, we identified Cys^377^ in SERCA (Figure 9E) and Cys^121^ in RyR1 (Figure 9F), the functions of which are sensitive to oxidation at cysteine residues (53–57). Previous studies have shown that SERCA Cys^377^ is one of 9 cysteines selectively oxidized during biological aging and sulfonylated in vitro by amino acid peroxides concomitantly with decreased activity in both cases (54, 55). Interestingly, RyR1 Cys^121^ is 1 of only 2 cysteines (out of 100 total) that were previously shown to be both oxidized and sulfenylated in vitro (58).

We also identified several cysteine residues with evidence of irreversible oxidation after ECC from the redox-sensitive contractile proteins Myh4 (Cys^1443^, Figure 9A), fMybpc (Cys^140^, Cys^143^, and Cys^1115^, Figure 9, B and C), and troponin I (Cys^134^, Figure 9D), which could explain the role of myofibrillar dysfunction in ECC force loss (59). Previously, increases in oxidative stress were shown to impair muscle force production by decreasing myofibrillar calcium sensitivity (51, 60, 61). Interestingly, oxidation of troponin I Cys^134^ led to suppression of calcium sensitivity that was reversed with DTT reduction and prevented by protective *S*-glutathionylation (62). Similarly, oxidation of myosin modulates contractile function (63), although a specific effect of Cys^1443^ oxidation on Myh4 activity has not been reported. Oxidative functional regulation of the fast isoform of Mybpc (fMybpc) in skeletal muscle is unknown, but oxidation-mediated regulation of the cardiac isoform is linked to contractile dysfunction, and given the structural similarity between isoforms, it is plausible that fMybpc function is similarly modulated by oxidation (64–68).

Our chemo-proteomic analysis also identified 2 redox proteins that may indirectly impact force production by perturbing protein thiol hyperoxidation (51). The activity of catalase, an H_2_O_2_-specific scavenger, can be suppressed by excess H_2_O_2_ and rescued by NaHS treatment (42, 69). Prx1 is another H_2_O_2_-specific scavenger that also functions as a redox relay that mediates H_2_O_2_ signaling (70–72). Inhibition of Prx1 is associated with dysregulated intercellular ROS levels and increased cellular oxidative stress (73), while *S*-nitrosylation or sulfonylation at Cys^173^ results in impaired Prx1 activity (74, 75). Interestingly, Prx2 levels are decreased in *mdx* skeletal muscle as a result of irreversible oxidation and proteolytic degradation, while forced overexpression of Prx2 partially protects *mdx* muscle from ECC force drop (6). Collectively, the functional breadth of proteins identified in our chemo-proteomic screen supports the concept that ECC force loss is not the consequence of a single point of failure in *mdx* muscle, but rather an accumulation of functional disruptions across multiple proteins involved in excitation contraction coupling.

A limitation of this study is the lack of data demonstrating that irreversible oxidation at the cysteines reported in Figure 9 impairs contractile protein function. However, many of the proteins identified in Figure 9 are extremely large and function within macromolecular complexes exposed to a complex dystrophic redox environment. Thus, detailed functional assessments will likely require new Cys-to-Ser knockin mouse lines crossed onto the *mdx* background, which is beyond the scope of the current study. In the meantime, we have shown that H_2_S transiently prevents *mdx* skeletal muscle from ECC force loss, one of the most robust and reproducible phenotypes of the dystrophin-deficient *mdx* mouse. Our data indicate that the mechanism of *mdx* ECC force loss is dependent on 3 factors: (a) increased oxidation of the thiol proteome at baseline, which compromises a key protective buffer against oxidative stress; (b) aberrant TSP activity that manifests in decreased H_2_S, which compromises a second important protective mechanism against irreversible thiol oxidation; and (c) excess ROS production during ECCs that leads to irreversible oxidation of susceptible proteins critical for muscle contraction. Our collective data provide a mechanistic framework that potentially explains how a variety of diverse interventions that target different aspects of the muscle contractile apparatus may act through a common redox-based pathway to prevent the inhibitory effects of irreversible cysteine oxidation and consequently *mdx* ECC force loss.

## Methods

### Sex as a biological variable.

Our study exclusively examined male mice as DMD is an X-linked recessive disorder that predominantly affects boys. It is unknown whether these findings are relevant to female mice.

### Animals.

All wild-type mice used in this study were on the C57BL/10SnJ background. All mice on the *mdx* background used the C57BL/10ScSn-Dmdmdx/J strain of *mdx* mice from The Jackson Laboratory. The Dys^ΔMTB^-*mdx* line expressed a skeletal muscle–specific, full-length dystrophin/utrophin chimera using the human skeletal actin (HSA) promoter, in which microtubule-binding spectrin repeats 20–24 of dystrophin were replaced by non-binding repeats 18–22 of utrophin (22). Mice with a genetic deletion of the NOX2 scaffolding subunit p47phox (p47^–/–^) were bred onto the *mdx* background (76) and obtained from George Rodney at the Baylor College of Medicine. Male mice aged 90–115 days were used for all experiments.

### In vitro EDL physiological experiments.

Mice were anesthetized with sodium pentobarbital (75–100 mg/kg body mass). EDL muscles were dissected and suspended in a 1.5 mL bath assembly filled with Krebs-Ringer bicarbonate buffer (Krebs) (119 mM NaCl, 5 mM KCl, 1 mM MgSO_4_, 1 mM KH_2_PO_4_, 10 mM glucose, 0.17 mM leucine, 0.1 mM isoleucine, 0.2 mM valine, 1.25 mM CaCl_2_, 0.10 U/mL insulin, 25 mM NaHCO_3_). Krebs was perfused with 95% O_2_ and maintained at 25°C. Muscles were secured to a 300B-LR dual-mode muscle lever system with 5-0 suture (model 300B-LR Dual-Mode Lever Arm, Aurora Scientific Inc.) and adjusted to anatomical optimal length (L_O_). Muscle ISO contractions were elicited with a stimulus for 200 milliseconds at 175 Hz and 150 V. Eccentric contractions (ECCs) were performed by passive shortening of the EDL to 95% L_O_ and then stimulation for 200 milliseconds while the muscle was simultaneously lengthened to 105% L_O_ at 0.5 L_O_/s. All experiments involving pharmacological treatments were either suspended directly in Krebs (20 mM NAC [Sigma-Aldrich, A7250], 40 mM l-cysteine [Sigma-Aldrich, C7352], 250 μM or 1,000 μM NaHS [Cayman Chemical, 10012555], 5 μM gp91ds-TAT [Anaspec, AS-63818], 0.55 mM H_2_O_2_ [Sigma-Aldrich, H1009]) or, if suspended in a vehicle, added to the bath at the start of the protocol (100 μM GYY4137 [Sigma-Aldrich, SML0100] in H_2_O, 50 μM GSK2795039 [GLPBIO, GC32681] in DMSO, and 0.5 mM 7-azido-4-methylcoumarin [Sigma-Aldrich, 802409] in DMSO). These pharmacological treatments did not have any acute effects on EDL muscle–specific ISO force production (Supplemental Figure 4). The same protocol was performed for all experiments unless otherwise stated: (a) a 10-minute equilibration, (b) 3 ISO contractions separated by 2 minutes, (c) a 15-minute incubation, (d) an isometric contraction followed by 2 minutes, and (e) 10 (or 20) ECCs separated by 3 minutes. Upon completion of the ECCs, EDLs were removed from the bath, weighed, flash-frozen, and stored at –80°C.

### Measurements of EDL force and ROS production.

Mice were anesthetized by isoflurane (2%) inhalation and euthanized by rapid cervical dislocation followed by thoracotomy. EDL muscles were surgically dissected fixed to a lever arm of a dual-mode lever system (Aurora Scientific Inc., 305C-LR-FP) using silk suture (4-0). The EDL was placed in a horizontal perfusion circulating bath containing Krebs (in mM): 2.0 CaCl_2_, 120.0 NaCl, 4.0 KCl, 1.0 MgSO_4_, 25.0 NaHCO_3_, 1.0 KH_2_PO_4_, 10.0 glucose, pH 7.3, and continuously gassed with 95% O_2_/5% CO_2_ at room temperature. Muscle length was adjusted to L_O_. The muscle was incubated for 30 minutes in 10 μM DCFH-DA (Sigma-Aldrich) in Krebs. During a 30-minute de-esterification period, NaHS (Cayman Chemical) Krebs was added to the bath at 16, 23, and 33 minutes to achieve a concentration of 250 μM, 500 μM, and 750 μM, respectively. The camera (MyoCam, Photometrics) was then focused on the muscle to view distinct fibers and set to record 1 frame every 5 seconds using MetaFluor (Molecular Devices). ECCs were performed as described above. Before the eccentric protocol was started, a 2-minute baseline was recorded. After each ECC, the focal plane was adjusted to maintain view of the fibers. After the protocol, 1 mM (final concentration) H_2_O_2_ was added to the bath, and fluorescence was recorded for 5 minutes. Average DCF fluorescence for 30 seconds prior to the next ECC was used to quantify oxidation of DCFH.

### Western blotting.

Whole gastrocnemius muscles were homogenized in lysis buffer (1% SDS in 1× PBS with protease inhibitors). Protein lysates were centrifuged for 10 minutes at 20,817g, and 40 μg of the soluble fraction was loaded on 10% polyacrylamide gel for 1 hour at 150 V and subsequently transferred to 0.45 μm polyvinylidene fluoride membrane for 1 hour at 0.8 A. Membranes were blocked with 5% nonfat milk in PBS for 30 minutes at room temperature. The following primary antibodies were incubated with the membranes overnight at 4°C: rabbit polyclonal anti-AE1 (Proteintech, 28131-1-AP; 1:500), rabbit polyclonal anti-ASCT1 (Sigma-Aldrich, AV43827; 1:1,000), rabbit polyclonal anti-ACY1 (Abcam, ab189399; 1:1,000), mouse monoclonal anti-GCL (Thermo Fisher Scientific, MA5-26346; 1:1,000), rabbit polyclonal anti-GSS (Thermo Fisher Scientific, PA5-97810; 1:1,000), rabbit polyclonal anti-GR (Thermo Fisher Scientific, PA570004; 1:1,000), rabbit polyclonal anti-GPX (Thermo Fisher Scientific, PA5-26323; 1:1,000), rabbit polyclonal anti-GRX (Abcam, ab45953; 1:1,000), rabbit polyclonal anti-GSTm1 (Sigma-Aldrich, AV41769; 1:1,000), rabbit polyclonal anti-GSTm2 (Thermo Fisher Scientific, PA5-89264; 1:1,000), rabbit polyclonal anti-CBS (Proteintech, 14787-1-AP; 1:1,000), rabbit polyclonal anti-CSE (Abcam, ab151769; 1:500), mouse monoclonal anti-GOT1 (Abcam, ab239487; 1:1,000), rabbit polyclonal anti-MPST (Novus Biologicals, NBP1-54734; 1:1,000), rabbit monoclonal anti-TRX (Abcam, ab273877; 1:1,000), mouse monoclonal anti-GAPDH (Sigma-Aldrich, G8795; 1:10,000), and rabbit polyclonal anti-GAPDH (Sigma-Aldrich, G9545; 1:10,000). Membranes were washed with 0.1% Tween in PBS and incubated in secondary antibodies (DyLight 680 Goat Anti-Mouse IgG [Cell Signaling Technology, 5470S], DyLight 680 Goat Anti-Rabbit IgG [Cell Signaling Technology, 5366S], DyLight 800 Goat Anti-Mouse IgG [Cell Signaling Technology, 5257S], and DyLight 800 Goat Anti-Rabbit IgG [Cell Signaling Technology, 5151S]) for 1 hour at room temperature. The membranes were imaged and analyzed with a LI-COR Odyssey imaging system (LI-COR Biotechnology) and associated software (Image Studio Lite v5.2).

### Glutathione assay.

Muscles were homogenized (1:10, muscle mass/volume) in a 1:1 solution consisting of 1× MES buffer (Cayman Chemical, item 703010) and MPA (Sigma-Aldrich, 239275) 10% (m/v) and subsequently centrifuged at 10,000*g* for 15 minutes at 4°C. The supernatant was extracted and divided into 2 fractions for separate assessments of total glutathione (GSH) and oxidized glutathione (GSSG). GSSG samples were treated with triethanolamine (TEAM) at 6 μL per 100 μL sample and 2-vinylpyridine (Sigma-Aldrich, 132292) at 2 μL per 100 μL sample for 1 hour at room temperature. Standards for GSH and GSSG samples were made in MES/MPA solution (range, 0–8 μM) with GSSG (Cayman Chemical, item 703014) and run in duplicate. Standards for GSSG samples were treated with TEAM and 2-vinylpyridine and incubated for 1 hour at room temperature. Standards or samples (20 μL) were loaded onto a clear plate, and 120 μL of a solution consisting of equal volumes of 1.7 mM 5,5-dithio-bis-(2-nitrobenzoic acid) (DTNB; Sigma-Aldrich, 22582) and glutathione reductase (10 U/mL; Sigma-Aldrich, G3664) was added to each sample. After a 30-second incubation, 60 μL of 0.8 mM β-nicotinamide adenine dinucleotide 2′-phosphate reduced tetrasodium salt hydrate (β-NADPH; Sigma-Aldrich, N1630) was added to each sample. A spectrophotometer (Spectra Max Plus, Molecular Devices) was used to read the plate at 412 nm every 15 seconds for 3 minutes. The rates of change in optical density from each sample and standard were used to calculate the concentration of total GSH per milligram of tissue. The ratio of GSSG to GSH was calculated as follows: (total GSH – GSSG)/GSSG.

### Quantification of protein thiol oxidation using maleimide.

Tibialis anterior or EDL muscles were homogenized (1:20, muscle mass/volume) in 20% (vol/vol) trichloroacetic acid (Sigma-Aldrich, T6399) and incubated for 1 hour at 4°C. After centrifugation (20,817*g*, 5 minutes, 4°C), samples were washed twice with acetone, resuspended in lysis buffer (1% SDS in 1× PBS with protease inhibitors), diluted to 1 mg/mL, and divided into 2 fractions. Both fractions were processed as follows: 5 mM 5,5-dimethyl-1,3-cyclohexanedione (Dimedone, Sigma-Aldrich, D153303) was added to samples for 1 hour at 37°C; trichloroacetic acid/acetone precipitation was performed (added equal volume of 20% trichloroacetic acid, incubated 10 minutes on ice, centrifuged at 20,817*g* for 5 minutes at 4°C, rinsed twice with acetone); fractions were resuspended in lysis buffer containing 50 mM *N*-ethylmaleimide (NEM; Sigma-Aldrich, E3876) for 30 minutes at 37°C; trichloroacetic acid/acetone precipitation was performed; fractions were resuspended in lysis buffer containing either 0 mM or 5 mM dithiothreitol (DTT; MilliporeSigma, DTT-RO) for 1 hour at 37°C; trichloroacetic acid/acetone precipitation was performed; and fractions were resuspended in lysis buffer containing 0.1 μM DyLight 800 maleimide (Thermo Fisher Scientific, 46621) for 2 hours at 37°C. Laemmli sample buffer was added to all samples (1:6, vol/vol) before SDS-PAGE was performed. Gels were imaged using LI-COR Odyssey (LI-COR Biotechnology), and protein thiol oxidation was quantified by subtraction of the non-DTT-treated sample signal from the DTT-treated sample signal and normalized by total protein assessed via Coomassie brilliant blue stain.

### Quantification of protein thiol sulfenylation using Dyn-2.

Gastrocnemius muscles were homogenized (1:20, muscle mass/volume) in 20% (vol/vol) trichloroacetic acid (Sigma-Aldrich, T6399) and incubated for 1 hour at 4°C. After centrifugation (20,817*g*, 5 minutes, 4°C), samples were washed twice with acetone, resuspended in lysis buffer (1% SDS in 1× PBS with protease inhibitors), diluted to 1 mg/mL, and divided into 2 fractions. Sample fractions were treated with or without or 5 mM (final concentration) Dyn-2 (Kerafast, EST018, or Cayman Chemical, 11220). Samples were incubated for 1 hour at 37°C. Dyn-2 was removed from the solution using a trichloroacetic acid/acetone precipitation. Dyn-2–labeled protein was linked with the IRDye 800CW Azide Infrared Dye (LI-COR Biotechnology, 929-60000) using click chemistry. A cocktail containing (in final sample concentrations) 1 mM Tris(2-carboxyethyl)phosphine hydrochloride solution (TCEP; Sigma-Aldrich, 646547), 100 μM Tris[(1-benzyl-1*H*-1,2,3-triazol-4-yl)methyl]amine (TBTA; Sigma-Aldrich, 678937), 0.3 μM azide dye, and 1 mM CuSO_4_ (added last; MilliporeSigma, PHR1477) was added to each sample and incubated at 37°C for 1 hour. EDTA (40 mM) was added to each sample to quench the reaction. Laemmli sample buffer was added to all samples (1:6, vol/vol) before SDS-PAGE was performed. Gels were imaged using LI-COR Odyssey (LI-COR Biotechnology), and protein thiol sulfenylation was quantified by subtraction of the non–Dyn-2–treated sample signal from the Dyn-2–treated sample signal and normalization by total protein assessed via Coomassie brilliant blue stain.

### H_2_S content assay.

H_2_S content was assessed as previously described (12). Muscles were homogenized in cold non-denaturing lysis buffer (50 mM Tris-HCl, pH 8.0, 150 mM NaCl, 2% Triton X-100). Protein lysates were centrifuged for 10 minutes at 20,817*g* at 4°C. The supernatant was removed, and protein concentration was determined by Pierce BCA Protein Assay Kit (Thermo Fisher Scientific, 23225). Three replicates of 150 μg of protein were loaded into a Corning flat, clear-bottom, black, 96-well plate, followed by the addition of 0.01 mM 7-azido-4-methylcoumarin (Sigma-Aldrich, 802409) to each well. The plates were sealed, wrapped in foil, and agitated at 200 rpm at 37°C for 1 hour. Plates were imaged on a fluorescence plate reader (excitation 365 nm, emission 450 nm).

### Muscle preparation for proteomics experiments.

EDL muscles from WT and *mdx* mice were collected after incubation in Krebs or the performing of 4 in vitro ECCs in the absence or presence of 750 μM NaHS, as detailed in the in vitro EDL physiology experimental method. EDL muscles were weighed and homogenized in buffer 1 (1% SDS, 200 mM Tris-HCl, pH 8.5, 5 mM EDTA, and protease inhibitors) at a 1:25 tissue-to-volume ratio using a bead blender (Next Advance, 4116-BBY24M). After centrifugation at 20,817*g* for 5 minutes at 4°C, protein concentration was measured by A_280_ absorbance, aliquoted into 2 fractions, (a) reversible cysteine oxidation and (b) total cysteine oxidation, and flash-frozen in liquid nitrogen to prevent spontaneous cysteine oxidation during sample preparation. Sample aliquots for reversible cysteine oxidation were incubated with 100 mM iodoacetamide in the dark for 30 minutes to chemically block free thiols and then precipitated with acetone and resuspended in buffer 2 (1% SDS, 200 mM Tris-HCl, pH 7.0, 5 mM EDTA, and protease inhibitors). Subsequently, sample aliquots for reversible and total cysteine oxidation determination were incubated with 20 mM TCEP (Thermo Fisher Scientific, 77720) for 1 hour in the dark to reduce all reversibly oxidized cysteine residues, followed by sample clean-up with Zeba desalting columns (Thermo Fisher Scientific, 89890), acetone precipitation, and resuspension in buffer 2. Protein concentration was reassessed with A_280_ absorbance, and all samples were diluted to 1 mg/mL. Liquid chromatography–mass spectrometry–grade methanol was added to iodoTMTsixplex Isobaric Label Reagent tubes (Thermo Fisher Scientific, 90101), and then sample was added to achieve a 10-fold excess of iodoTMT reagent over free thiols. Samples were incubated for 1 hour at 37°C in the dark, quenched with 20 mM DTT, precipitated with acetone, and resuspended to 1 mg/mL in 50 mM ammonium bicarbonate (pH 8.0) before undergoing protein digestion (1:50, wt/wt, trypsin/protein ratio). Samples were acidified with 10% TFA and dried overnight by vacuum centrifugation. Labeled peptide samples were resuspended with 1× TBS, pH 7.4, and applied to spin columns with immobilized anti-TMT antibody resin (Thermo Fisher Scientific, 90076) to enrich samples for iodoTMT-labeled peptides. Samples were frozen and dried down overnight by vacuum centrifugation, then resuspended in 98:2 (vol/vol) water/acetonitrile with 0.2% formic acid for liquid chromatography–tandem mass spectrometry (LC-MS/MS) proteomics analysis.

### Cysteine oxidation chemo-proteomics experiments.

Chemo-proteomics experiments were performed at the University of Minnesota in collaboration with the Center for Metabolomics and Proteomics (CMSP). All data were collected on an Orbitrap Eclipse mass spectrometer coupled to a Dionex Ultimate 3000 RSLCnano LC pump. Peptides were separated using a 109-minute gradient at 0.315–0.350 μL/min on a C18-AQ ReproSil-Pur column measuring 300 mm with an internal diameter of 100 μm, 1.9 μm particle size, and 120 Å pore size (Maisch GmbH Ammerbuch). Buffer A consisted of water with 0.1% (vol/vol) formic acid, and buffer B consisted of acetonitrile with 0.1% (vol/vol) formic acid. High-field asymmetric-waveform ion mobility spectroscopy (FAIMS) was enabled during experimental acquisition with the following compensation voltage settings: –45 V, –60 V, and –75 V. Voltage was kept at 2.1 kV for positive ion mode, and the ion transfer tube temperature was set to 275°C. At the MS1 stage, the mass spectrometer scanned masses in the range of 400–1,400 *m*/*z* at a resolution of 120K with an AGC of 4.0 × 10^5^ over a 50-millisecond maximal injection time. At the MS2 stage, ions were fragmented by collisional dissociation with a collision of 40% at a detector resolution of 50K with an AGC of 1 × 10^5^ over a 200-millisecond maximal injection time, and the Fourier transform first mass mode was fixed at 110 *m*/*z*.

### Proteomics data analysis.

The proteomic raw mass spectra data were analyzed with Proteome Discoverer 2.5.0.400 (Thermo Fisher Scientific) by CMSP, and output files were generated for each 6-plex within each screen with proteins and respective peptide quantification. Custom routines were created in R project (version 2023.06.0+421) to perform data filtering, clean-up, and statistical analysis. First, common peptide sequences were determined across the five 6-plexes within each screen; then the common peptide sequences across screens 1 and 2 were determined. The ratio of peptide abundance from the oxidized channel to peptide abundance from the total channel was calculated using raw peptide abundances from each group (screen 1: WT, WT+ECC, *mdx*; screen 2: *mdx*, *mdx*+ECC, *mdx*+ECC+NaHS). Before statistical analysis, oxidized/total ratios for each peptide were normalized between both screens using the common *mdx* control group. For the group comparisons shown in Figure 6, *P* values were calculated using Student’s *t* test between the means of the normalized oxidized/total ratios of each group. Log_2_ fold changes and –log_10_ *P* values were calculated to generate volcano plots (Figure 6).

### Statistics.

Statistical analysis was performed using GraphPad Prism software (version 9 or 10). All data are presented as mean ± SEM. Statistical significance was determined by 2-tailed Student’s *t* test, 1-way ANOVA, 2-way ANOVA, or 2-way repeated-measures ANOVA. Tukey’s post hoc tests were performed for 1- and 2-way ANOVAs, while Šidák’s post hoc tests were performed for 2-way repeated-measures ANOVAs. Significance was defined as *P* less than 0.05.

### Study approval.

Animal housing, treatment, experiments, and euthanasia were performed in accordance with NIH guidelines and approved by the Institutional Animal Care and Use Committee of the University of Minnesota or Baylor College of Medicine.

### Data availability.

Proteomic data generated and used in this study are available at MassIVE Repository (accession MSV000092827; https://doi.org/doi:10.25345/C5KW57V2Q). All original code has been deposited at https://doi.org/10.7910/DVN/XMNGZ0 and is publicly available. Any additional information required to reanalyze the data reported in this paper is available upon request. All figure data are available in the Supporting Data Values file.

## Author contributions

WMS, EEJ, and JME conceptualized the study. WMS, EKF, CLC, and JME performed data curation and formal analysis. All authors performed investigation. JME, GGR, and WMS acquired funding. WMS and JME performed visualization. JME supervised the study. WMS and JME wrote the original draft of the article. WMS, EEJ, KSF, CLC, DAL, GGR, and JME reviewed and edited the manuscript.

## Supplementary Material

Supplemental data

Unedited blot and gel images

Supporting data values

## Version 1 01/14/2025

In-Press Preview

## Version 2 03/03/2025

Electronic publication
