# Supplementary material for: Impaired hydrogen sulfide biosynthesis underlies eccentric contraction–induced force loss in dystrophin-deficient skeletal muscle
Source: J Clin Invest. 2025 Jan 14;135(5):e176942. doi: 10.1172/JCI176942 (PMC11870723; doi:10.1172/JCI176942)
Supplement: Supplemental data [file jci-135-176942-s168.pdf]

Table S1 – Summary and Categorization of publications involving ECC force loss in *mdx* mice

Author	Year	Title	Implicated Contractile Element	Muscle	Method
Krag et al.[1]	2004	"Heregulin ameliorates the dystrophic phenotype in mdx mice."	Utrophin	EDL	*in vitro*
Blaauw et al.[2]	2008	"Akt activation prevents the force drop induced by eccentric contractions in dystrophin-deficient skeletal muscle."	Utrophin / Redox Homeostasis	GAS	*in vivo* / *in vitro*
Amenta et al.[3]	2011	"Biglycan recruits utrophin to the sarcolemma and counters dystrophic pathology in mdx mice"	Utrophin	EDL	*in vitro*
Selsby et al.[4]	2012	"Rescue of dystrophic skeletal muscle by PGC-1α involves a fast to slow fiber type shift in the mdx mouse."	Utrophin	EDL	*in vitro*
Al-Rewashdy et al.[5]	2015	"Utrophin A is essential in mediating the functional adaptations of mdx mouse muscle following chronic AMPK activation."	Utrophin	EDL	*in vitro*
Gibbs et al.[6]	2016	"High levels of sarcospan are well tolerated and act as a sarcolemmal stabilizer to address skeletal muscle and pulmonary dysfunction in DMD."	Utrophin	EDL	*in vitro*
Trajanovska et al. [7]	2019	"Muscle specific kinase protects dystrophic mdx mouse muscles from eccentric contraction-induced loss of force-producing capacity."	Utrophin	TA	*in situ*
Pratt et al.[8]	2013	"Effects of in vivo injury on the neuromuscular junction in healthy and dystrophic muscles."	Excitability	Quad	*in vivo*
Call et al.[9]	2013	"Acute failure of action potential conduction in mdx muscle reveals new mechanism of contraction-induced force loss."	Excitability	TA	*in vivo*
Roy et al.[10]	2016	"Dystrophin restoration therapy improves both the reduced excitability and the force drop induced by lengthening contractions in dystrophic mdx skeletal muscle."	Excitability	TA / EDL	*in situ*
Baumann et al.[11]	2020	"Plasmalemma Function Is Rapidly Restored in *Mdx* Muscle after Eccentric Contractions."	Excitability	TA	*in vivo*
Whitehead et al.[12]	2006	"Streptomycin reduces stretch-induced membrane permeability in muscles from mdx mice."	Calcium Signaling	EDL	*in vitro*
Bellinger et al.[13]	2009	"Hypernitrosylated ryanodine receptor calcium release channels are leaky in dystrophic muscle."	Calcium Signaling	EDL	*in situ*
Zanou et al.[14]	2009	"Essential role of TRPV2 ion channel in the sensitivity of dystrophic muscle to eccentric contractions."	Calcium Signaling	EDL	*in vitro*
Morine et al.[15]	2010	"Overexpression of SERCA1a in the mdx diaphragm reduces susceptibility to contraction-induced damage."	Calcium Signaling	DIA	*in vitro*
Han et al.[16]	2011	"Dystrophin deficiency exacerbates skeletal muscle pathology in dysferlin-null mice."	Calcium Signaling	EDL	*in vitro*
Mázala et al.[17]	2015	"SERCA1 overexpression minimizes skeletal muscle damage in dystrophic mouse models."	Calcium Signaling	Quad	*in vivo*
Rader et al.[18]	2016	"Role of dystroglycan in limiting contraction-induced injury to the sarcomeric cytoskeleton of mature skeletal muscle."	Redox Homeostasis / Calcium Signaling	EDL	*in vitro*
Ward et al.[19]	2018	"GsMTx4-D provides protection to the D2.mdx mouse."	Calcium Signaling	GAS	*in vivo*
García-Castañeda et al.[20]	2022	"Postdevelopmental knockout of Orai1 improves muscle pathology in a mouse model of Duchenne muscular dystrophy."	Calcium Signaling	EDL	*in vitro*
Lin et al.[21]	2022	"Pharmacological TRPC6 inhibition improves survival and muscle function in mice with Duchenne muscular dystrophy"	Calcium Signaling	GAS	*in vivo*
Whitehead et al.[22]	2008	"N-Acetylcysteine ameliorates skeletal muscle pathophysiology in mdx mice."	Redox Homeostasis	EDL	*in vitro*
Whitehead et al.[23]	2010	"Skeletal muscle NADPH oxidase is increased and triggers stretch-induced damage in the mdx mouse."	Redox Homeostasis	FDB	*in vitro*
Baltgalvis et al.[24]	2011	"Transgenic overexpression of γ-cytoplasmic actin protects against eccentric contraction-induced force loss in mdx mice."	Redox Homeostasis	EDL	*in vitro*
Khairallah et al.[25]	2012	"Microtubules underlie dysfunction in duchenne muscular dystrophy."	Redox Homeostasis	GAS/FDB	*in vivo* / *in vitro*
Moorwood et al.[26]	2014	"Caspase-12 ablation preserves muscle function in the mdx mouse."	Redox Homeostasis	EDL	*in vitro*

Ismail et al.[27]	2014	"Diapocynin, a dimer of the NADPH oxidase inhibitor apocynin, reduces ROS production and prevents force loss in eccentrically contracting dystrophic muscle."	Redox Homeostasis	EDL	*in vitro*
Rebolledo et al.[28]	2016	"Sarcolemmal targeting of nNOSμ improves contractile function of mdx muscle."	Redox Homeostasis	TA	*in situ*
Marrocco et al.[29]	2017	"Pharmacological Inhibition of PKCθ Counteracts Muscle Disease in a Mouse Model of Duchenne Muscular Dystrophy."	Redox Homeostasis	EDL	*in vitro*
Lindsay et al.[30]	2018	"Xanthine oxidase is hyper-active in Duchenne muscular dystrophy."	Redox Homeostasis	EDL	*in vitro*
Vitiello et al.[31]	2018	"Drug Repurposing for Duchenne Muscular Dystrophy: The Monoamine Oxidase B Inhibitor Safinamide Ameliorates the Pathological Phenotype in mdx Mice and in Myogenic Cultures From DMD Patients."	Redox Homeostasis	GAS	*in vivo*
Olthoff et al.[32]	2018	"Loss of peroxiredoxin-2 exacerbates eccentric contraction-induced force loss in dystrophin-deficient muscle."	Redox Homeostasis	EDL	*in vitro*
Demonbreun et al.[33]	2021	"Anti-latent TGFβ binding protein 4 antibody improves muscle function and reduces muscle fibrosis in muscular dystrophy."	Redox Homeostasis	TA	*in situ*
Bonato et al.[34]	2023	"Cyclin D3 deficiency promotes a slower, more oxidative skeletal muscle phenotype and ameliorates pathophysiology in the mdx mouse model of Duchenne muscular dystrophy."	Redox Homeostasis	EDL	*in situ*
Blaauw et al.[35]	2010	"Eccentric contractions lead to myofibrillar dysfunction in muscular dystrophy."	Myofibrillar dysfunction	GAS	*in vivo* / *in vitro*
Baumann et al.[36]	2022	"Mechanisms of weakness in *mdx* muscle following *in vivo* eccentric contractions."	Calcium Signaling / Myofibrillar dysfunction	TA / EDL	*in vivo* / *in vitro*
Russell et al.[37]	2023	"Modulating fast skeletal muscle contraction protects skeletal muscle in animal model of Duchenne muscular dystrophy."	Myofibrillar dysfunction	EDL	*in vitro*

Abbreviations: EDL: extensor digitorum longus, SOL: soleus, GAS: gastrocnemius, FDB: flexor digitorum longus, TA: tibialis anterior, Quad: quadriceps.

Table S2 - Cysteine residues of interest from proteins related to muscle contraction or redox homeostasis

Master Protein Name	Master Protein Accession	Gene ID	Peptide Sequence [Position in Master]	Cysteine Position
Sodium/potassium-transporting ATPase subunit alpha	D3YYN7	*Atp1a2*	NI**C**FFSTN**C**VEGTAR [239-253]	241; 247
Sarcoplasmic/endoplasmic reticulum calcium ATPase 1 (SERCA)	Q8R429	*Atp2a1*	ANA**C**NSVIR [468-476]	471
	Q8R429	*Atp2a1*	EVTGSIQL**C**R [606-615]	614
	Q8R429	*Atp2a1*	SMSVY**C**SPAK [493-502]	498
	Q8R429	*Atp2a1*	VDGDV**C**SLNEFSITGSTYAPEGEVLK [372-397]	377
Catalase	P24270	*Cat*	LGPNYLQIPVN**C**PYR [366-380]	377
Aspartate aminotransferase, cytoplasmic	P05201	*Got1*	DIRPY**C**YWDAEK [155-166]	160
Myosin-binding protein C, slow-type	A0A571BEN1	*Mybpc1*	AVNDLGTVEIE**C**K [1090-1102]	1101
	A0A571BEN1	*Mybpc1*	VFAENM**C**GLSEDATMTK [974-990]	980
Myosin-binding protein C, fast-type	Q5XKE0	*Mybpc2*	DVCDS**C**SFNVDVEAPR [138-153]	140
	Q5XKE0	*Mybpc2*	**C**FTELFVK [330-337]	330
	Q5XKE0	*Mybpc2*	**C**TLADDAAYEVAVQDEK [313-329]	313
	Q5XKE0	*Mybpc2*	RPSPFDAGTYS**C**R [1104-1116]	1115
Myosin-4	Q5SX39	*Myh4*	SNAA**C**AALDK [1439-1448]	1443
Myosin light chain kinase 2	Q8VCR8	*Mylk2*	VEVGQAL**C**LTAR [255-266]	262
Myomesin-3	A2ABU4	*Myom3*	ELIEL**C**SGR [144-152]	149
Myotilin	Q9JIF9	*Myot*	HFIQPQNP**C**GSR [9-20]	17
Parkinson disease protein 7	Q99LX0	*Park7*	VTVAGLAGKDPVQ**C**SR [33-48]	46
Peroxiredoxin-1	P35700	*Prdx1*	HGEV**C**PAGWKPGSDTIKPDVNK [169-190]	173
Ryanodine receptor 1	E9PZQ0	*Ryr1*	**C**SN**C**YMVWGGDFVSPGQQGR [1490-1509]	1490; 1493
	E9PZQ0	*Ryr1*	GYPDIGWNP**C**GGER [2318-2331]	2327
	E9PZQ0	*Ryr1*	MYLS**C**LTTSR [117-126]	121
Troponin I, fast skeletal muscle	P13412	*Tnni2*	HKV**C**MDLR [131-138]	134
Thioredoxin	P10639	*Txn*	**C**MPTFQFYK [73-81]	73

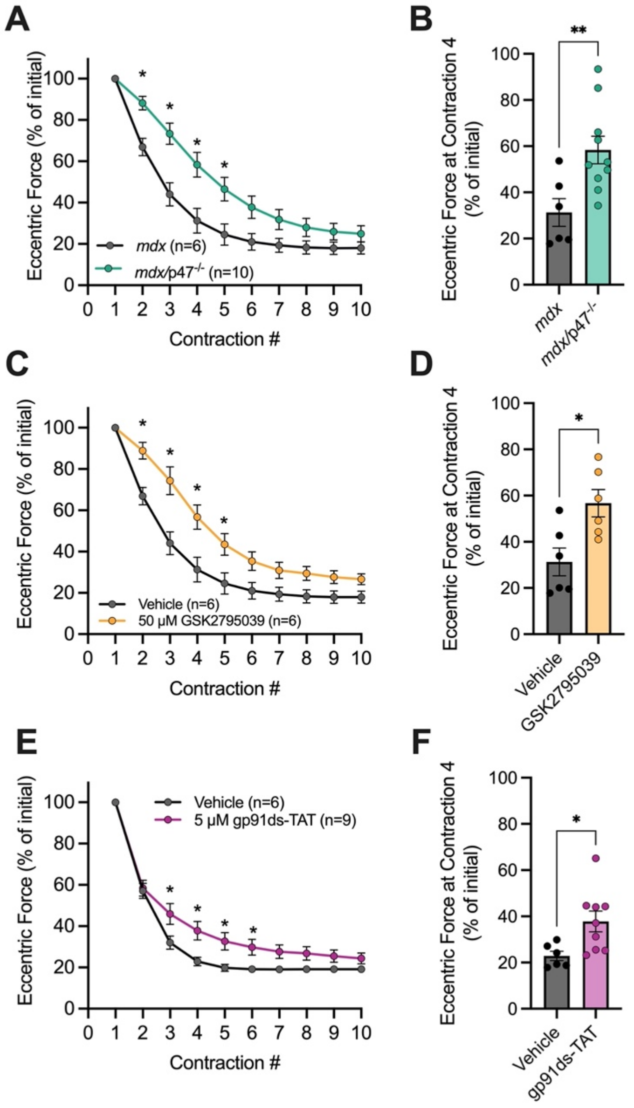

Fig. S1. Genetic ablation or pharmacologic inhibition of Nox2 partially protects *mdx* muscle from ECC force loss. (A and B): (A) Change in eccentric force during 10 ECCs in EDL muscles isolated from *mdx*/p47$^{-/-}$ and littermate control mice. **(B)** Eccentric force produced at ECC #4 in panel **(A)** showing individual data distribution for each genotype. **(C and E): (C)** Change in eccentric force during 10 ECCs in *mdx* EDL and incubated with vehicle or the NOX2 inhibitors **(C)** 50µM GSK2795039 or **(E)** 5µM gp91ds-TAT. **(D)**, **(F)** Eccentric force produced at ECC #4 from panels **(C)** and **(E)**, respectively, showing individual data distribution for each treatment condition. All ECC force data in **(A-C)** are expressed as a percentage of the force generated during the first contraction. Results are presented as mean ± SEM. *p<0.05, **p<0.01 by Student's t-test in **(B)**, **(D)**, and **(F)**, *p<0.05, ***p<0.001, or *p<0.05 by two-way repeated measures ANOVA in **(A)**, **(C)**, and **(E)**.

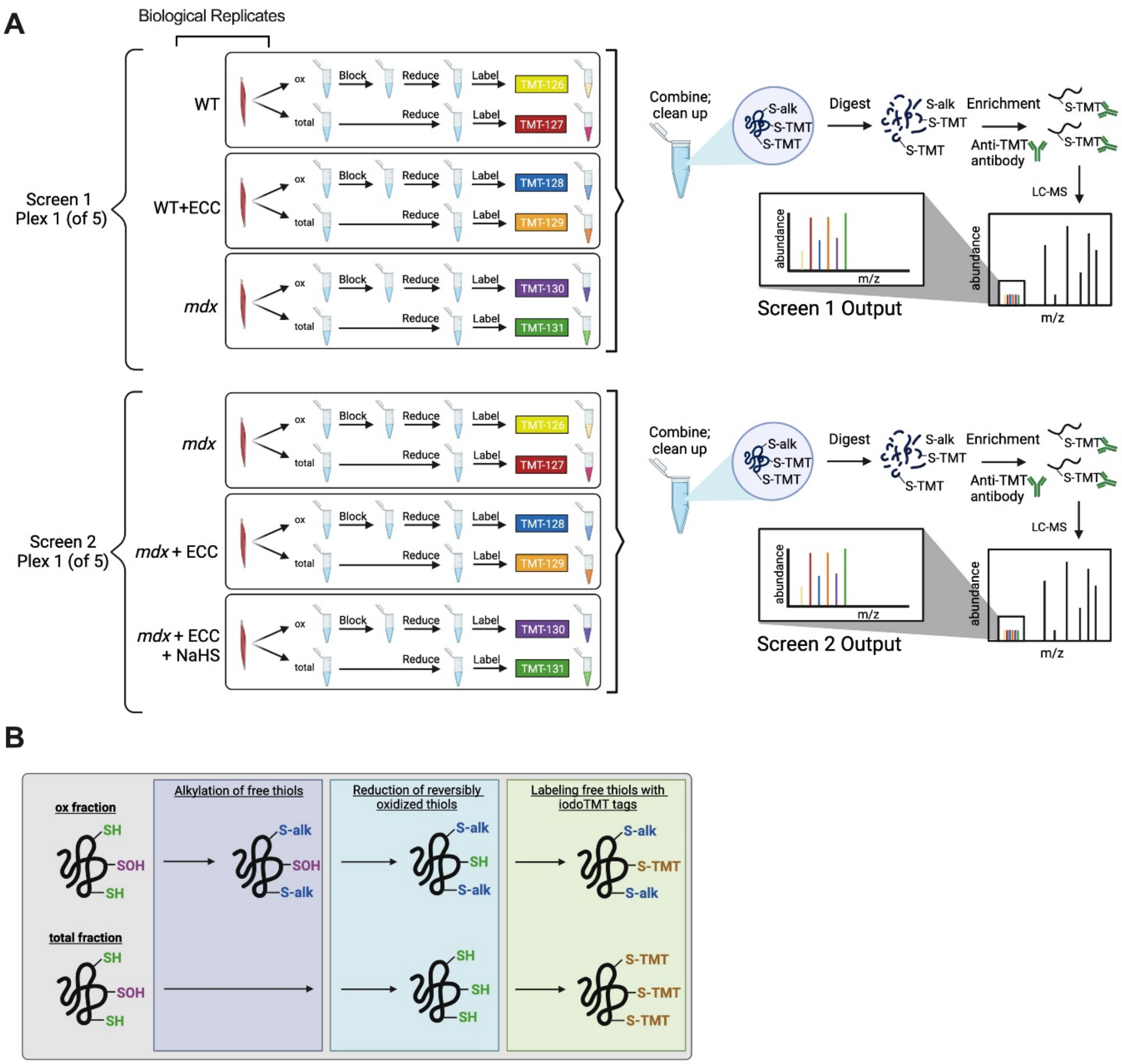

Fig. S2. Illustration of the methodology used for chemo-proteomic sample preparation. **(A)** Following ECCs, EDLs from all groups were flash frozen and stored in -80C. Frozen EDLs were homogenized and divided into two fractions: oxidized (ox) and total. Screen 1 a 5 plex assay with 6 channels in each plex containing ox and total samples from WT control, WT+ECC, and *mdx* control EDLs. Screen 2 was identical to screen 1 except for the samples which were *mdx*, *mdx*+ECC, and *mdx*+ECC+NaHS. Free thiols were blocked/alkylated in the ox fraction. Reversibly oxidized thiols were reduced, and all free thiols were labeled with iodoTMT tags. Protein samples were digested and enriched for TMT-labeled peptides. Samples were processed using liquid chromatography/tandem mass spectrometry (LC-MS/MS) and relative levels of oxidation between groups for a given cysteine were calculated. **(B)** Schematic showing additional details of the blocking/alkylating, reducing, and labeling processing steps for ox and total fractions.

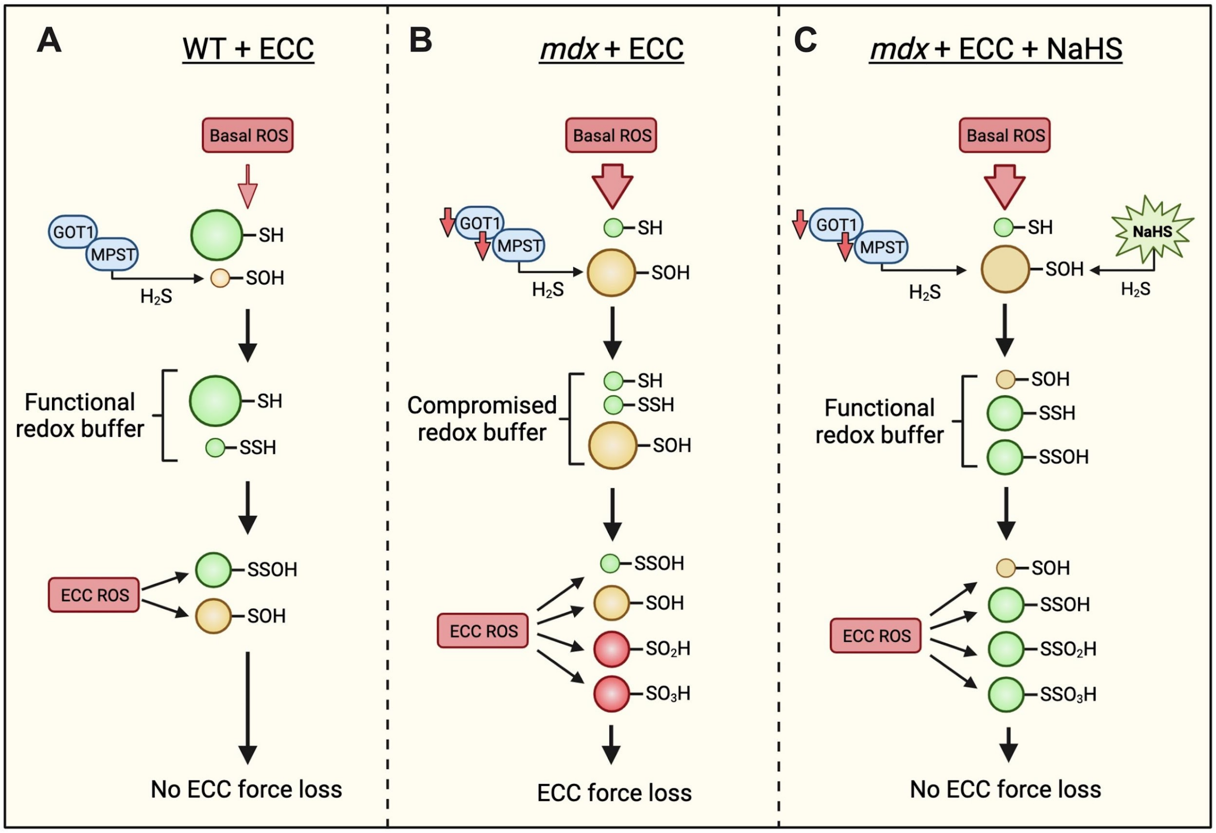

Fig. S3. Model illustrating the three elements driving ECC force loss in *mdx* skeletal muscle.

(A) Wild type (WT) skeletal muscle has a largely reduced proteome (large green circle) at baseline due to minimal basal reactive oxygen species (ROS) production. This, combined with normal levels of hydrogen sulfide (H_2S), preserves the function of the cysteine proteome redox buffer through persulfidation, allowing adequate buffering of excess ROS during ECCs. **(B)** Dystrophin-deficient (*mdx*) muscle has elevated levels of ROS and baseline proteome oxidation, which is compounded by a lack of persulfidiation-mediated protection due to a deficit in H_2S. As a result, *mdx* muscle has a compromised redox buffer that is overwhelmed with excess ROS during ECCs ultimately leading to irreversible oxidation and loss of protein function and muscle force. **(C)** Treatment of *mdx* muscles with NaHS effectively bypasses the defective H_2S-producing enzymes GOT1 and MPST and protects the redox buffer from irreversible oxidation, thereby preserving protein function and ECC force. Created with BioRender.com. Green circles = reduced (-SH) or persulfide-protected thiol (-SSH, SSOH, etc.); Yellow circles = oxidized thiol (-SOH); red circles = irreversibly oxidized thiol (-SO_2H, -SO_3H).

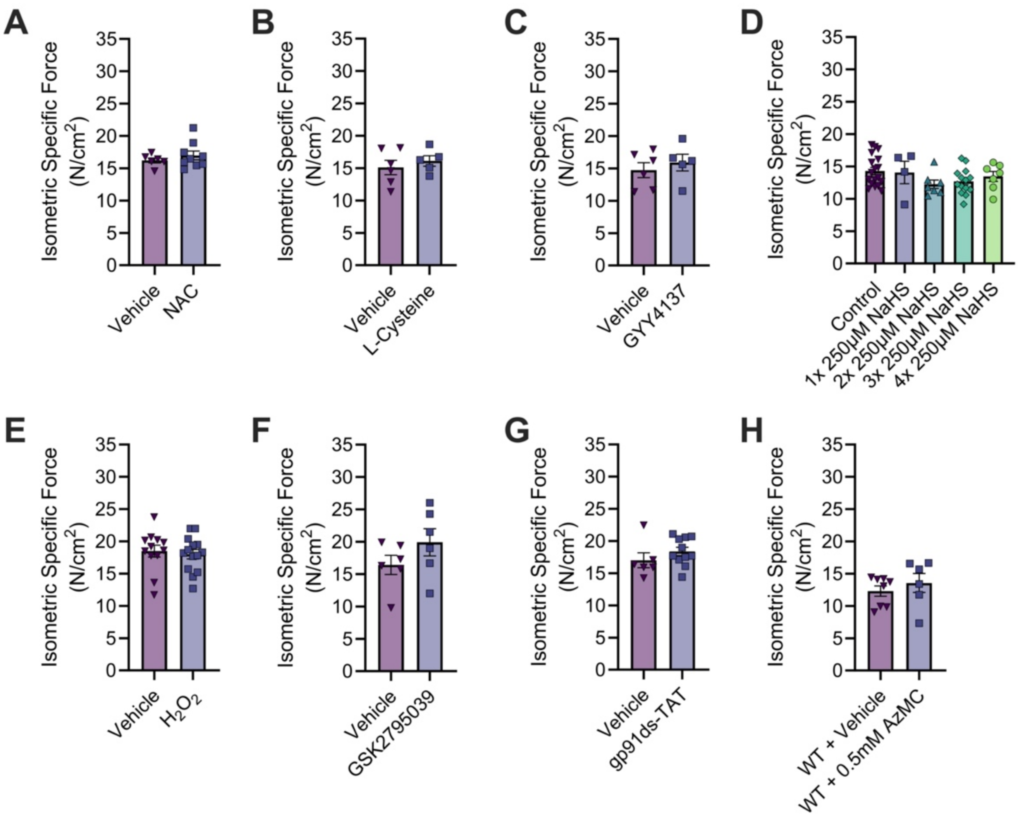

Fig. S4. Effects of various *in vitro* pharmacological treatments on ~~*mdx*~~ EDL muscle isometric specific force production.

(A-H) EDL muscles were isolated from *mdx* mice, suspended in an *in vitro* bath, and incubated with **(A)** vehicle or 20mM N-Acetyl Cysteine (NAC), **(B)** vehicle or 40mM L-Cysteine, **(C)** vehicle or 100µM GYY4137, **(D)** vehicle, 1x, 2x, 3x, or 4x doses of 250µM NaHS, **(E)** vehicle or 0.5mM H_2O_2, **(F-G)** vehicle or the NOX2 inhibitors **(F)** 50µM GSK2795039 or **(G)** 5µM gp91ds-TAT, **(H)** vehicle or 0.5mM of the H_2S scavenger 7-Azido-4-methylcoumarin (AzMC) Results are presented as mean ± SEM.

SUPPLEMENTAL REFERENCES

1. Krag, T.O.B., Bogdanovich, S., Jensen, C.J., Fischer, M.D., Hansen-Schwartz, J., Javazon, E.H., Flake, A.W., Edvinsson, L., and Khurana, T.S. (2004). Heregulin ameliorates the dystrophic phenotype in *mdx* mice. Proc. Natl. Acad. Sci. U.S.A. *101*, 13856–13860. 10.1073/pnas.0405972101.

2. Blaauw, B., Mammucari, C., Toniolo, L., Agatea, L., Abraham, R., Sandri, M., Reggiani, C., and Schiaffino, S. (2008). Akt activation prevents the force drop induced by eccentric contractions in dystrophin-deficient skeletal muscle. Human Molecular Genetics *17*, 3686–3696. 10.1093/hmg/ddn264.

3. Amenta, A.R., Yilmaz, A., Bogdanovich, S., McKechnie, B.A., Abedi, M., Khurana, T.S., and Fallon, J.R. (2011). Biglycan recruits utrophin to the sarcolemma and counters dystrophic pathology in mdx mice. Proc. Natl. Acad. Sci. U.S.A. *108*, 762–767. 10.1073/pnas.1013067108.

4. Selsby, J.T., Morine, K.J., Pendrak, K., Barton, E.R., and Sweeney, H.L. (2012). Rescue of Dystrophic Skeletal Muscle by PGC-1α Involves a Fast to Slow Fiber Type Shift in the mdx Mouse. PLoS ONE *7*, e30063. 10.1371/journal.pone.0030063.

5. Al-Rewashdy, H., Ljubicic, V., Lin, W., Renaud, J.-M., and Jasmin, B.J. (2015). Utrophin A is essential in mediating the functional adaptations of mdx mouse muscle following chronic AMPK activation. Human Molecular Genetics *24*, 1243–1255. 10.1093/hmg/ddu535.

6. Gibbs, E.M., Marshall, J.L., Ma, E., Nguyen, T.M., Hong, G., Lam, J.S., Spencer, M.J., and Crosbie-Watson, R.H. (2016). High levels of sarcospan are well tolerated and act as a sarcolemmal stabilizer to address skeletal muscle and pulmonary dysfunction in DMD. Hum. Mol. Genet., ddw356. 10.1093/hmg/ddw356.

7. Trajanovska, S., Ban, J., Huang, J., Gregorevic, P., Morsch, M., Allen, D.G., and Phillips, W.D. (2019). Muscle specific kinase protects dystrophic *mdx* mouse muscles from eccentric contraction-induced loss of force-producing capacity. J Physiol *597*, 4831–4850. 10.1113/JP277839.

8. Pratt, S.J.P., Shah, S.B., Ward, C.W., Inacio, M.P., Stains, J.P., and Lovering, R.M. (2013). Effects of *in vivo* injury on the neuromuscular junction in healthy and dystrophic muscles: Neuromuscular junction morphology. The Journal of Physiology *591*, 559–570. 10.1113/jphysiol.2012.241679.

9. Call, J.A., Warren, G.L., Verma, M., and Lowe, D.A. (2013). Acute failure of action potential conduction in mdx muscle reveals new mechanism of contraction-induced force loss. The Journal of physiology *591*, 3765–3776. 10.1113/jphysiol.2013.254656.

10. Roy, P., Rau, F., Ochala, J., Messéant, J., Fraysse, B., Lainé, J., Agbulut, O., Butler-Browne, G., Furling, D., and Ferry, A. (2016). Dystrophin restoration therapy improves both the reduced excitability and the force drop induced by lengthening contractions in dystrophic mdx skeletal muscle. Skeletal Muscle *6*, 23. 10.1186/s13395-016-0096-4.

11. Baumann, C.W., Warren, G.L., and Lowe, D.A. (2020). Plasmalemma Function Is Rapidly Restored in Mdx Muscle after Eccentric Contractions. Medicine & Science in Sports & Exercise *52*, 354–361. 10.1249/MSS.0000000000002126.

12. Whitehead, N.P., Streamer, M., Lusambili, L.I., Sachs, F., and Allen, D.G. (2006). Streptomycin reduces stretch-induced membrane permeability in muscles from mdx mice. Neuromuscular Disorders *16*, 845–854. 10.1016/j.nmd.2006.07.024.

13. Bellinger, A.M., Reiken, S., Carlson, C., Mongillo, M., Liu, X., Rothman, L., Matecki, S., Lacampagne, A., and Marks, A.R. (2009). Hypernitrosylated ryanodine receptor calcium release channels are leaky in dystrophic muscle. Nature Medicine *15*, 325–330. 10.1038/nm.1916.

14. Zanou, N., Iwata, Y., Schakman, O., Lebacq, J., Wakabayashi, S., and Gailly, P. (2009). Essential role of TRPV2 ion channel in the sensitivity of dystrophic muscle to eccentric contractions. FEBS Letters *583*, 3600–3604. 10.1016/j.febslet.2009.10.033.

15. Morine, K.J., Sleeper, M.M., Barton, E.R., and Sweeney, H.L. (2010). Overexpression of SERCA1a in the *mdx* Diaphragm Reduces Susceptibility to Contraction-Induced Damage. Human Gene Therapy *21*, 1735–1739. 10.1089/hum.2010.077.

16. Han, R., Rader, E.P., Levy, J.R., Bansal, D., and Campbell, K.P. (2011). Dystrophin deficiency exacerbates skeletal muscle pathology in dysferlin-null mice. Skeletal Muscle *1*, 35. 10.1186/2044-5040-1-35.

17. Mázala, D.A.G., Pratt, S.J.P., Chen, D., Molkentin, J.D., Lovering, R.M., and Chin, E.R. (2015). SERCA1 overexpression minimizes skeletal muscle damage in dystrophic mouse models. American Journal of Physiology-Cell Physiology *308*, C699–C709. 10.1152/ajpcell.00341.2014.

18. Rader, E.P., Turk, R., Willer, T., Beltrán, D., Inamori, K., Peterson, T.A., Engle, J., Prouty, S., Matsumura, K., Saito, F., et al. (2016). Role of dystroglycan in limiting contraction-induced injury to the sarcomeric cytoskeleton of mature skeletal muscle. Proc. Natl. Acad. Sci. U.S.A. *113*, 10992–10997. 10.1073/pnas.1605265113.

19. Ward, C.W., Sachs, F., Bush, E.D., and Suchyna, T.M. (2018). GsMTx4-D provides protection to the D2.mdx mouse. Neuromuscular Disorders *28*, 868–877. 10.1016/j.nmd.2018.07.005.

20. García-Castañeda, M., Michelucci, A., Zhao, N., Malik, S., and Dirksen, R.T. (2022). Postdevelopmental knockout of Orai1 improves muscle pathology in a mouse model of Duchenne muscular dystrophy. Journal of General Physiology *154*, e202213081. 10.1085/jgp.202213081.

21. Lin, B.L., Shin, J.Y., Jeffreys, W.P.D., Wang, N., Lukban, C.A., Moorer, M.C., Velarde, E., Hanselman, O.A., Kwon, S., Kannan, S., et al. (2022). Pharmacological TRPC6 inhibition improves survival and muscle function in mice with Duchenne muscular dystrophy. JCI Insight *7*, e158906. https://doi.org/10.1172/jci.insight.158906.

22. Whitehead, N.P., Pham, C., Gervasio, O.L., and Allen, D.G. (2008). N-Acetylcysteine ameliorates skeletal muscle pathophysiology in mdx mice. The Journal of physiology *586*, 2003–2014. 10.1113/jphysiol.2007.148338.

23. Whitehead, N.P., Yeung, E.W., Froehner, S.C., and Allen, D.G. (2010). Skeletal Muscle NADPH Oxidase Is Increased and Triggers Stretch-Induced Damage in the mdx Mouse. PLoS ONE *5*, e15354. 10.1371/journal.pone.0015354.

24. Baltgalvis, K.A., Jaeger, M.A., Fitzsimons, D.P., Thayer, S.A., Lowe, D.A., and Ervasti, J.M. (2011). Transgenic overexpression of γ-cytoplasmic actin protects against eccentric contraction-induced force loss in mdx mice. Skeletal muscle *1*, 32. 10.1186/2044-5040-1-32.

25. Khairallah, R.J., Shi, G., Sbrana, F., Prosser, B.L., Borroto, C., Mazaitis, M.J., Hoffman, E.P., Mahurkar, A., Sachs, F., Sun, Y., et al. (2012). Microtubules Underlie Dysfunction in Duchenne Muscular Dystrophy. Sci. Signal. *5*. 10.1126/scisignal.2002829.

26. Moorwood, C., and Barton, E.R. (2014). Caspase-12 ablation preserves muscle function in the mdx mouse. Human Molecular Genetics *23*, 5325–5341. 10.1093/hmg/ddu249.

27. Ismail, H.M., Scapozza, L., Ruegg, U.T., and Dorchies, O.M. (2014). Diapocynin, a Dimer of the NADPH Oxidase Inhibitor Apocynin, Reduces ROS Production and Prevents Force Loss in Eccentrically Contracting Dystrophic Muscle. PLoS ONE *9*, e110708. 10.1371/journal.pone.0110708.

28. Rebolledo, D.L., Kim, M.J., Whitehead, N.P., Adams, M.E., and Froehner, S.C. (2016). Sarcolemmal targeting of nNOSμ improves contractile function of *mdx* muscle. Hum. Mol. Genet. *25*, 158–166. 10.1093/hmg/ddv466.

29. Marrocco, V., Fiore, P., Benedetti, A., Pisu, S., Rizzuto, E., Musarò, A., Madaro, L., Lozanoska-Ochser, B., and Bouché, M. (2017). Pharmacological Inhibition of PKCθ Counteracts Muscle Disease in a Mouse Model of Duchenne Muscular Dystrophy. EBioMedicine *16*, 150–161. 10.1016/j.ebiom.2017.01.001.

30. Lindsay, A., McCourt, P.M., Karachunski, P., Lowe, D.A., and Ervasti, J.M. (2018). Xanthine oxidase is hyper-active in Duchenne muscular dystrophy. Free Radical Biology and Medicine *129*, 364–371. 10.1016/j.freeradbiomed.2018.10.404.

31. Vitiello, L., Marabita, M., Sorato, E., Nogara, L., Forestan, G., Mouly, V., Salviati, L., Acosta, M., Blaauw, B., and Canton, M. (2018). Drug Repurposing for Duchenne Muscular Dystrophy: The Monoamine Oxidase B Inhibitor Safinamide Ameliorates the Pathological Phenotype in mdx Mice and in Myogenic Cultures From DMD Patients. Front. Physiol. *9*, 1087. 10.3389/fphys.2018.01087.

32. Olthoff, J.T., Lindsay, A., Abo-Zahrah, R., Baltgalvis, K.A., Patrinostro, X., Belanto, J.J., Yu, D.-Y., Perrin, B.J., Garry, D.J., Rodney, G.G., et al. (2018). Loss of peroxiredoxin-2 exacerbates eccentric contraction-induced force loss in dystrophin-deficient muscle. Nature Communications. 10.1038/s41467-018-07639-3.

33. Demonbreun, A.R., Fallon, K.S., Oosterbaan, C.C., Vaught, L.A., Reiser, N.L., Bogdanovic, E., Velez, M.P., Salamone, I.M., Page, P.G.T., Hadhazy, M., et al. (2021). Anti-latent TGFβ binding protein 4 antibody improves muscle function and reduces muscle fibrosis in muscular dystrophy. Sci. Transl. Med. *13*, eabf0376. 10.1126/scitranslmed.abf0376.

34. Bonato, A., Raparelli, G., Luvisetto, S., Forconi, F., Cosentino, M., Tirone, F., Rizzuto, E., and Caruso, M. (2023). Cyclin D3 deficiency promotes a slower, more oxidative skeletal muscle phenotype and ameliorates pathophysiology in the *mdx* mouse model of Duchenne muscular dystrophy. The FASEB Journal *37*, e23025. 10.1096/fj.202201769R.

35. Blaauw, B., Agatea, L., Toniolo, L., Canato, M., Quarta, M., Dyar, K.A., Danieli-Betto, D., Betto, R., Schiaffino, S., and Reggiani, C. (2010). Eccentric contractions lead to myofibrillar dysfunction in muscular dystrophy. Journal of Applied Physiology *108*, 105–111. 10.1152/japplphysiol.00803.2009.

36. Baumann, C.W., Ingalls, C.P., and Lowe, D.A. (2022). Mechanisms of weakness in Mdx muscle following in vivo eccentric contractions. J Muscle Res Cell Motil *43*, 63–72. 10.1007/s10974-022-09617-1.

37. Russell, A.J., DuVall, M., Barthel, B., Qian, Y., Peter, A.K., Newell-Stamper, B.L., Hunt, K., Lehman, S., Madden, M., Schlachter, S., et al. (2023). Modulating fast skeletal muscle contraction protects skeletal muscle in animal models of Duchenne muscular dystrophy. Journal of Clinical Investigation *133*, e153837. 10.1172/JCI153837.
