# Supplementary material for: Impaired hydrogen sulfide biosynthesis underlies eccentric contraction–induced force loss in dystrophin-deficient skeletal muscle
Source: J Clin Invest. 2025 Jan 14;135(5):e176942. doi: 10.1172/JCI176942 (PMC11870723; doi:10.1172/JCI176942)
Supplement: Unedited blot and gel images [file jci-135-176942-s169.pdf]

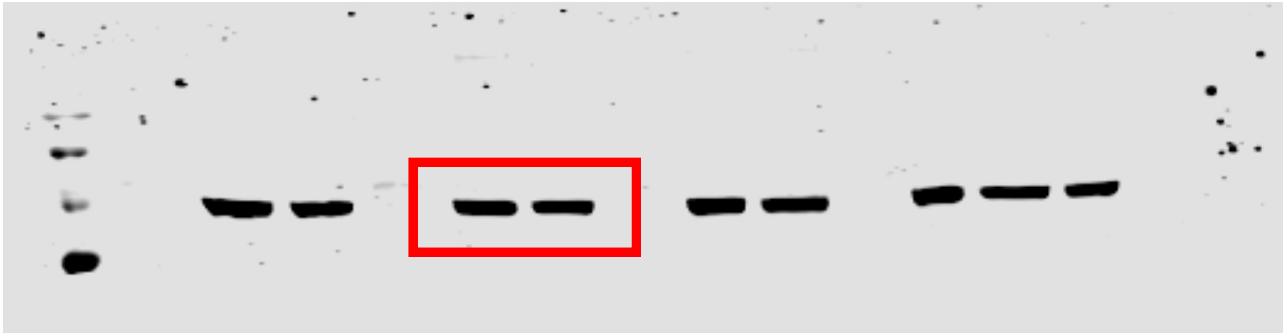

Full unedited gel for Figure 1C – AE1 (GAPDH loading control on next page)

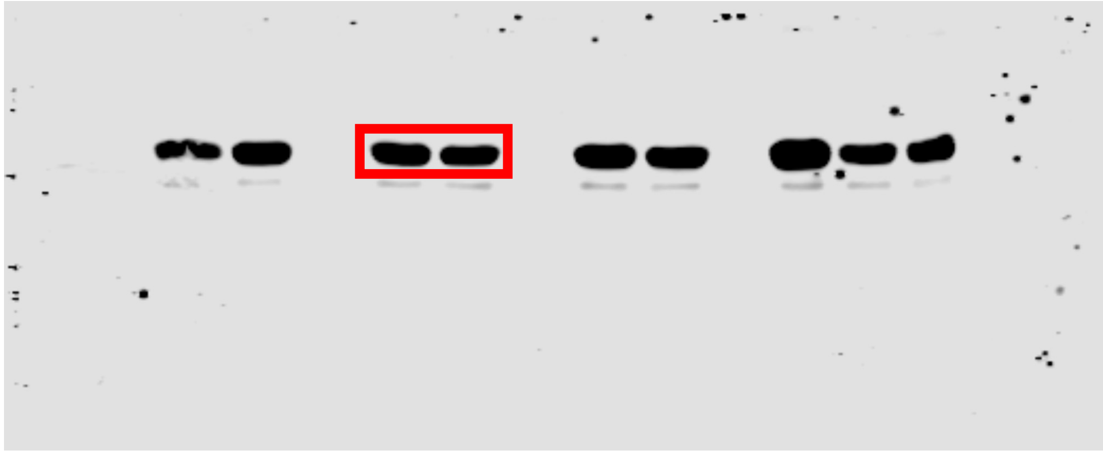

ASCT1

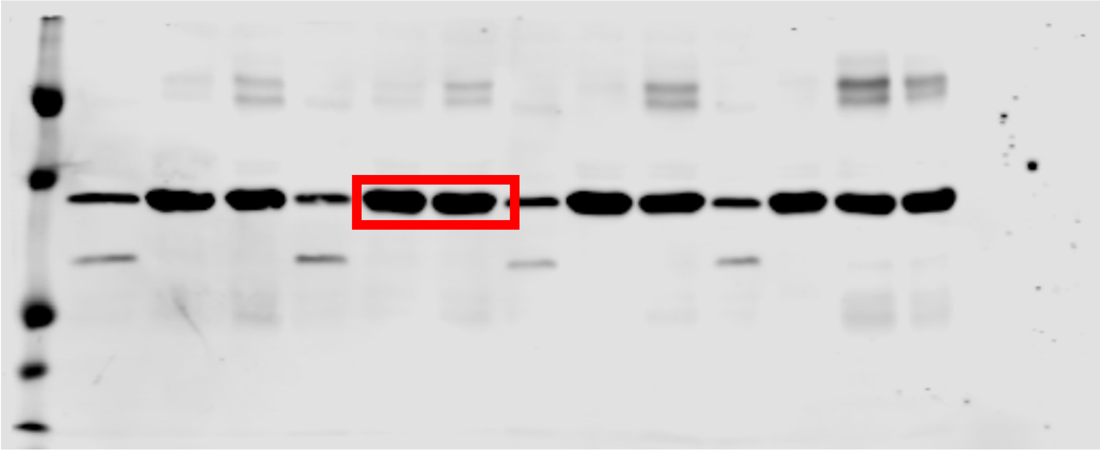

GAPDH (image used as representative for loading control)

Full unedited gel for Figure 1C – ASCT1 and GAPDH

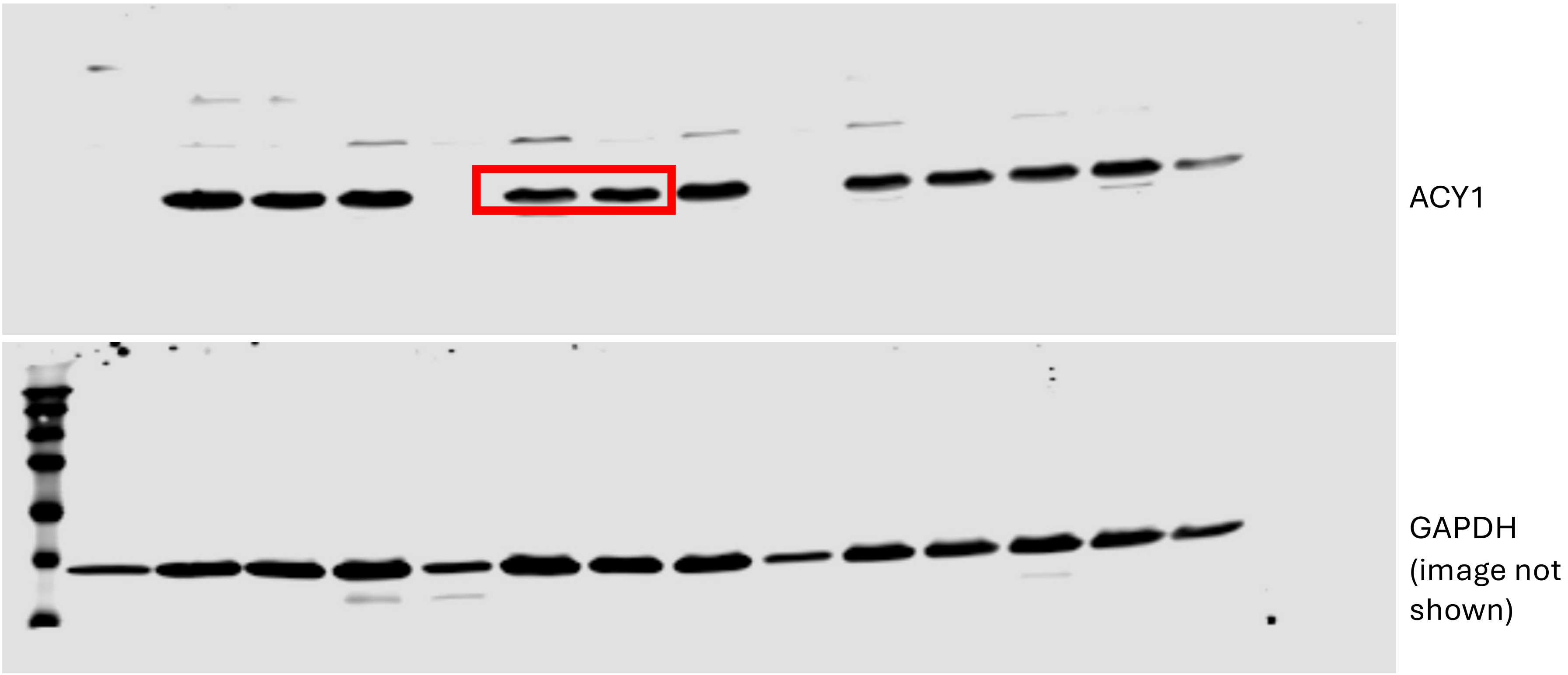

Full unedited gel for Figure 1C – ACY1

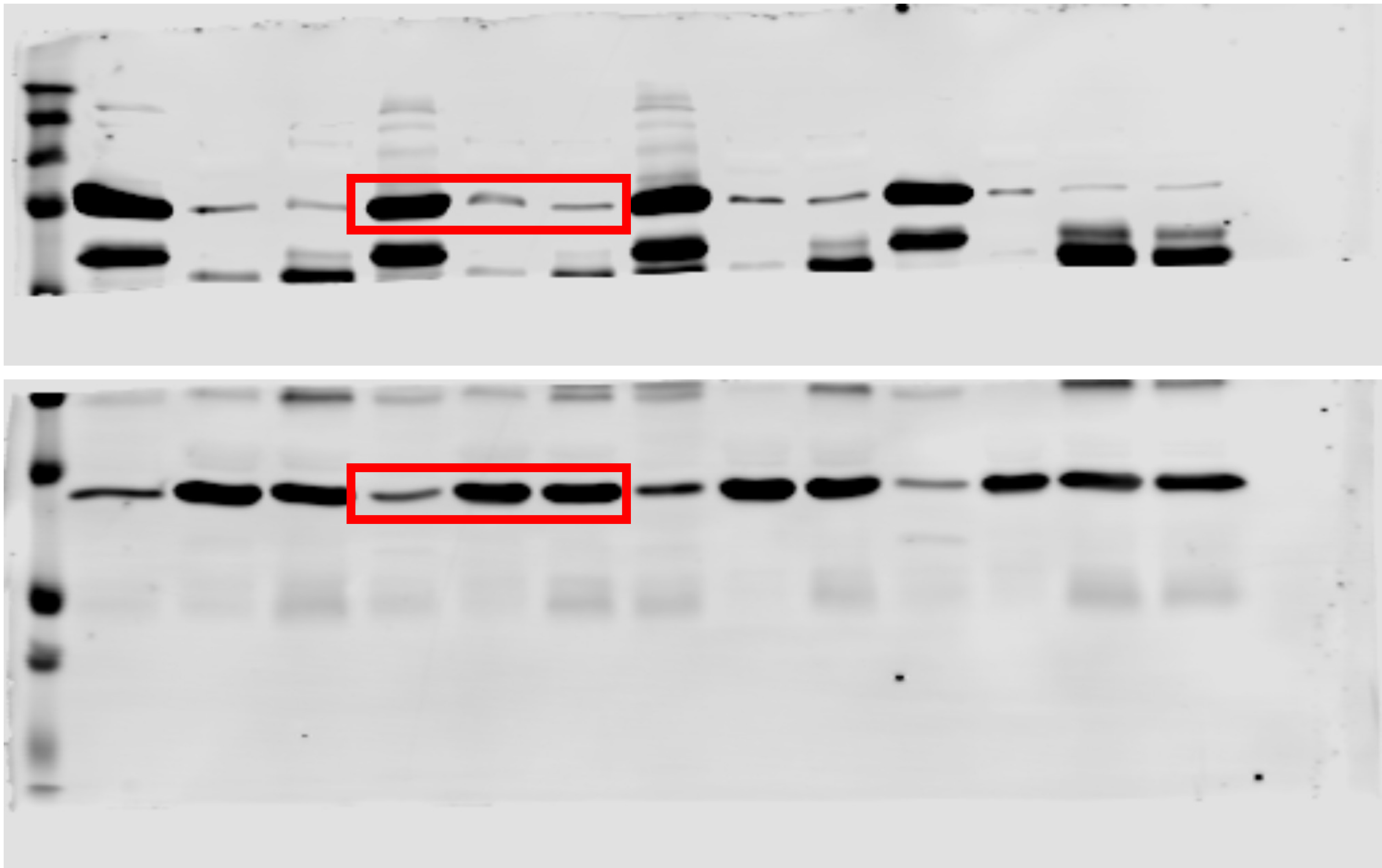

GCL

GAPDH (image used as representative for loading control)

Full unedited gel for Figure 2A – GCL and GAPDH

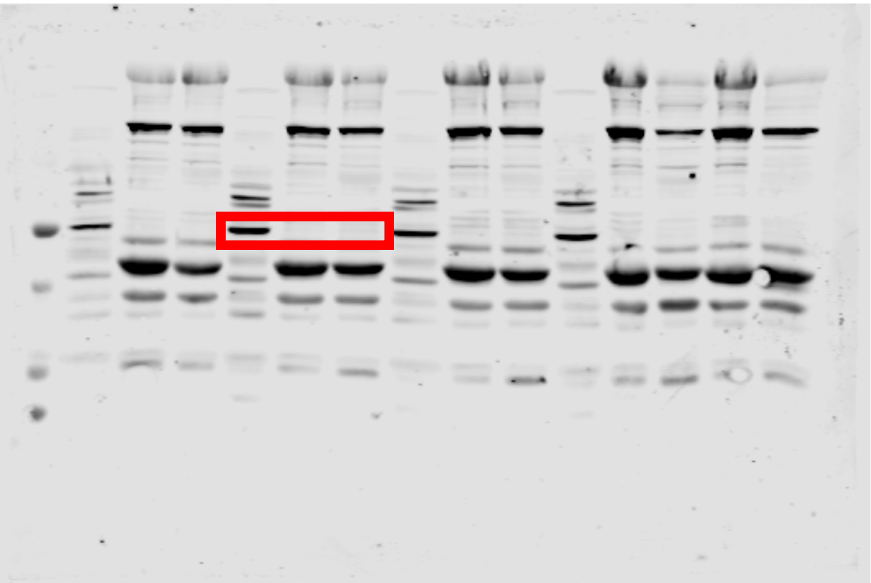

GS

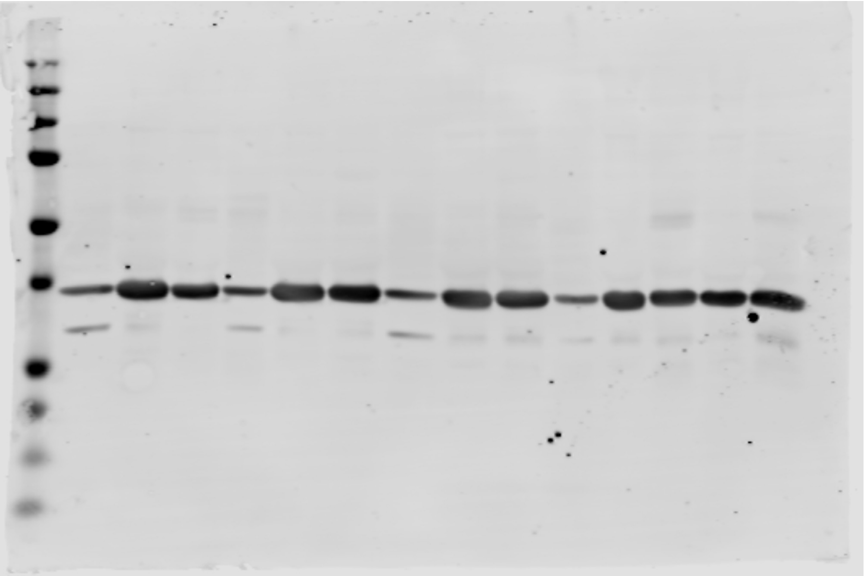

GAPDH (image not shown)

Full unedited gel for Figure 2A – GS

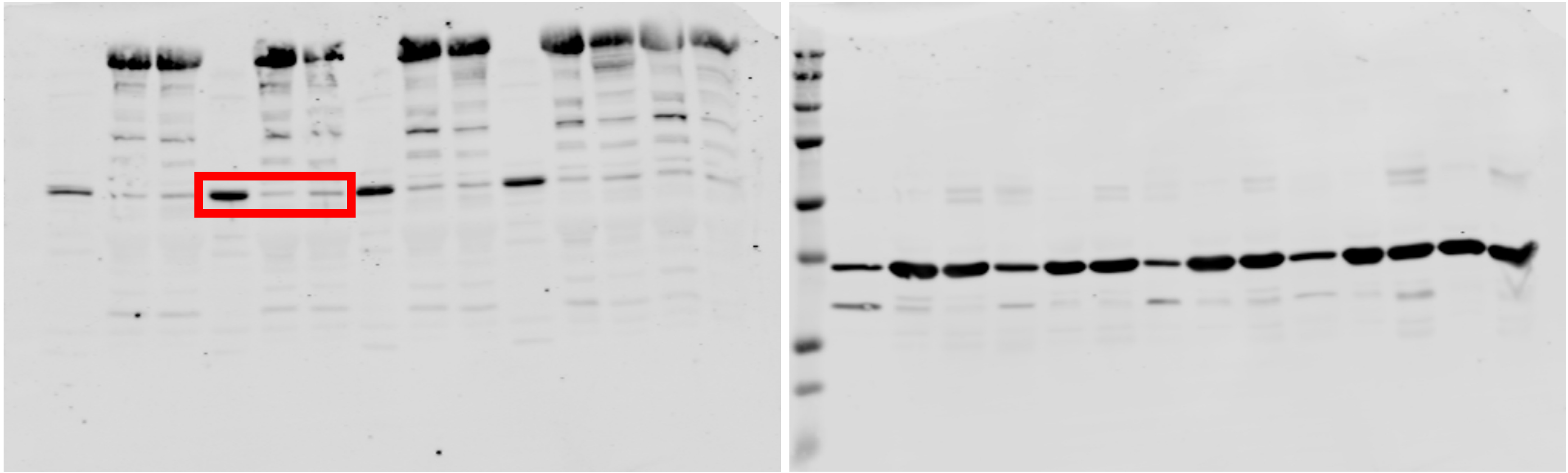

Full unedited gel for Figure 2A – GR and GAPDH

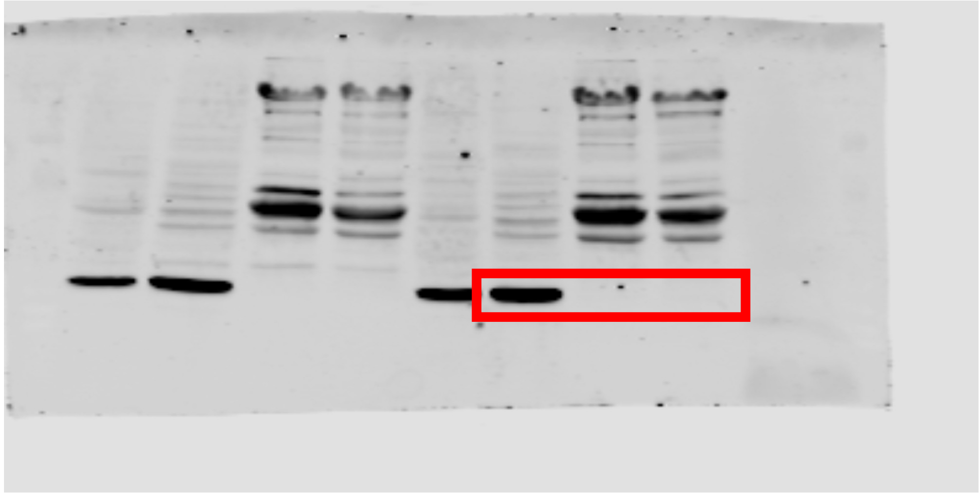

GPX

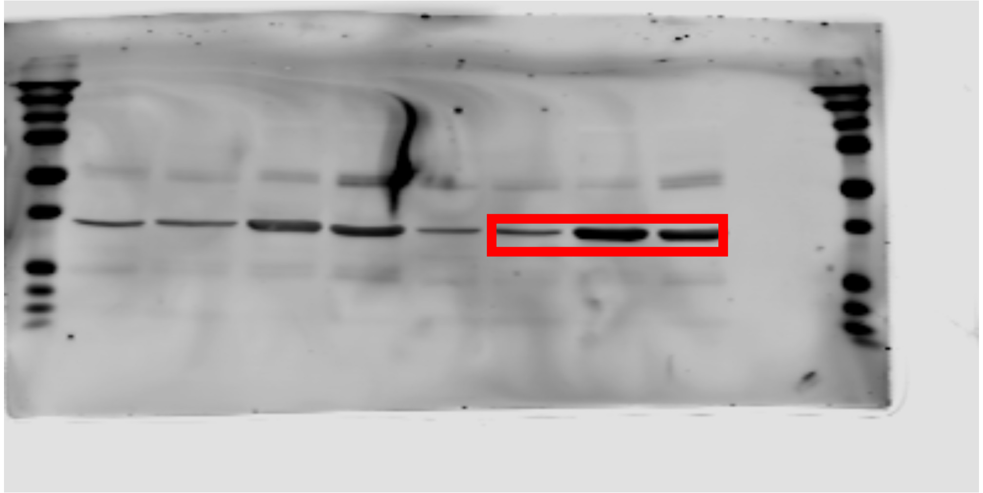

GAPDH (image used as representative for loading control)

Full unedited gel for Figure 2A – GPX and GAPDH

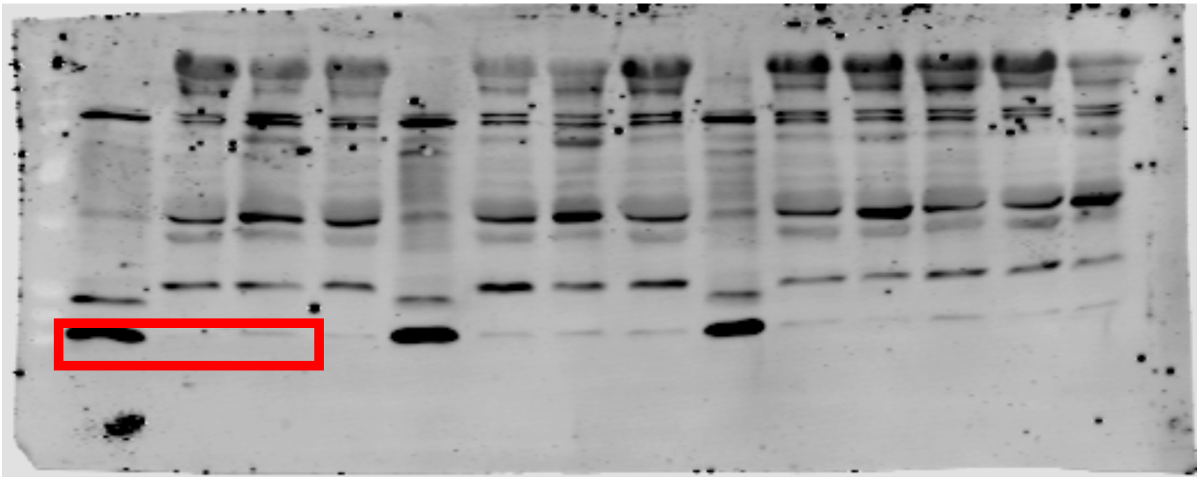

GRX

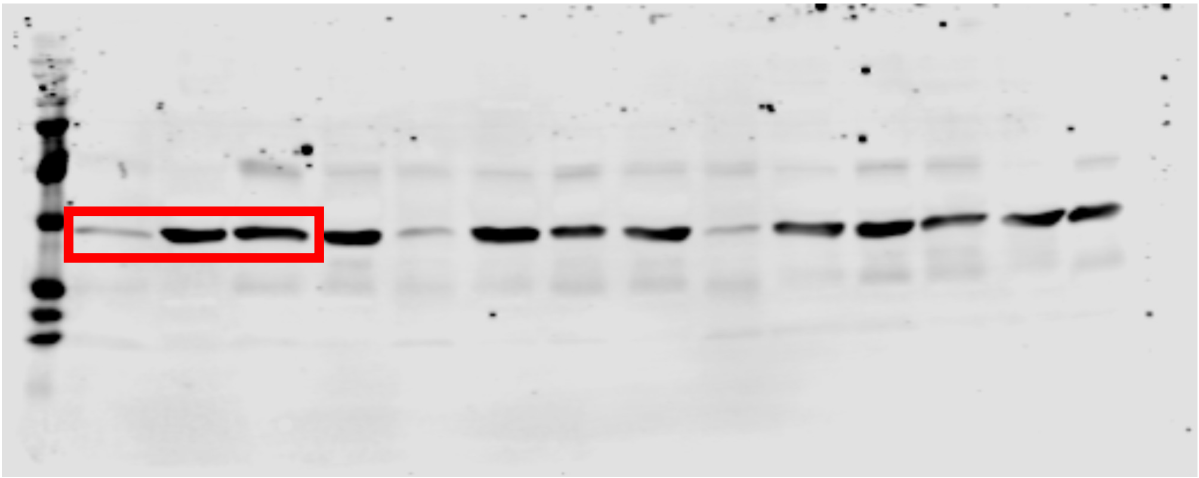

GAPDH (image used as representative for loading control)

Full unedited gel for Figure 2A – GRX

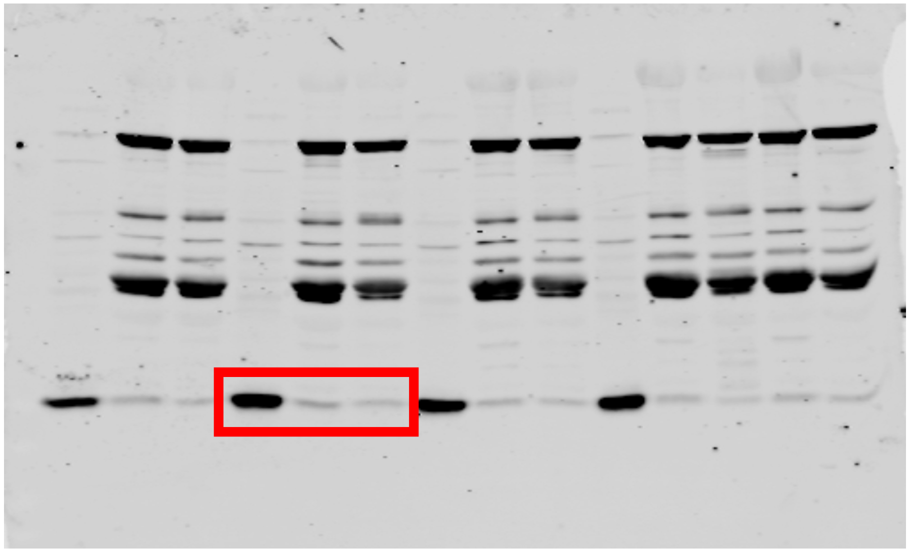

GSTm1

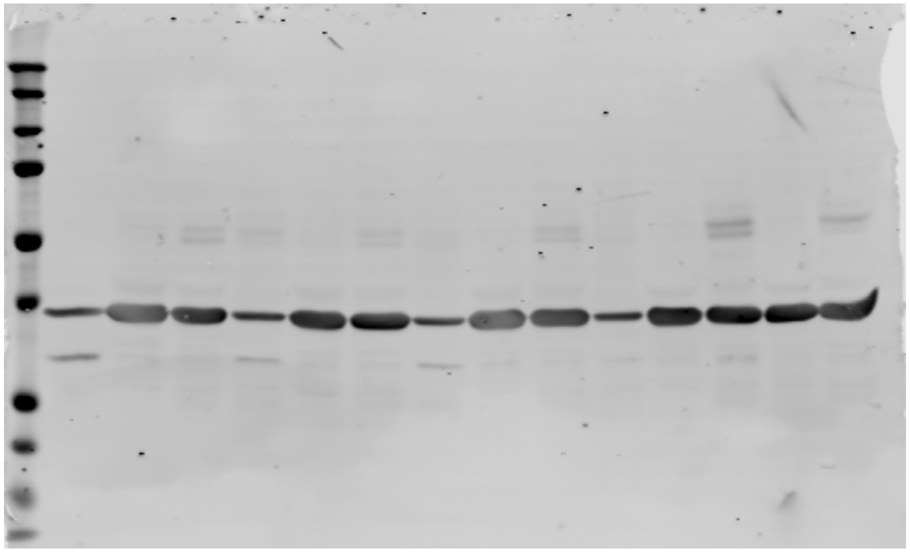

GAPDH (image not shown)

Full unedited gel for Figure 2A – GSTm1 and GAPDH

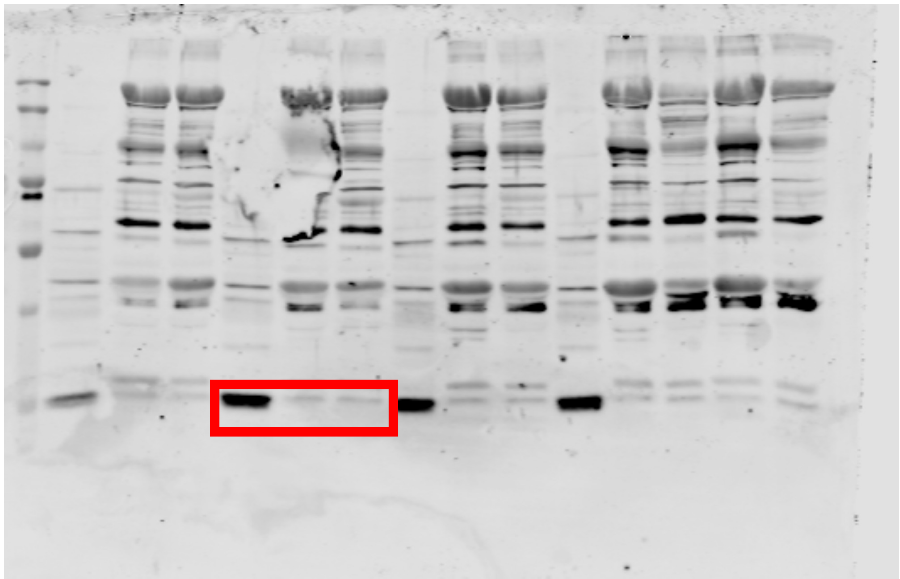

GSTm2

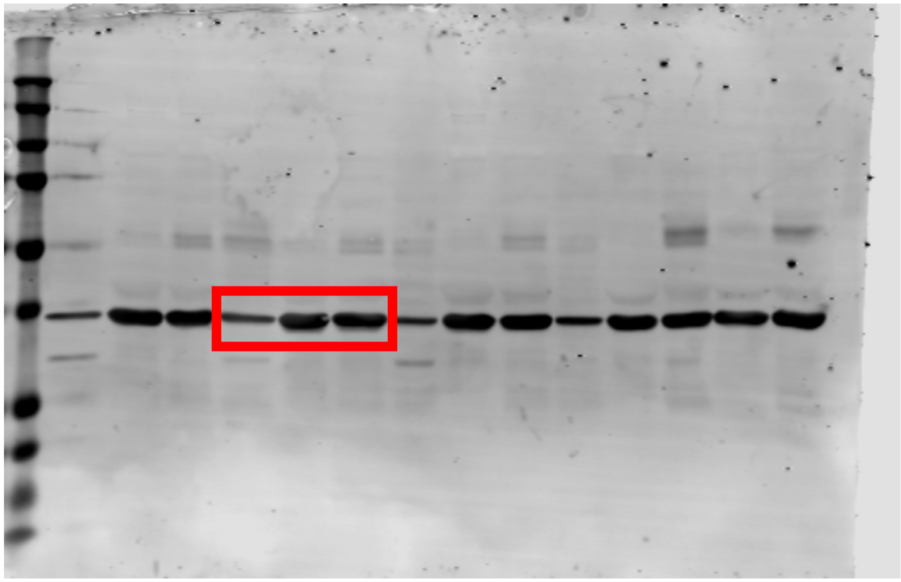

GAPDH (image used as representative for loading control)

Full unedited gel for Figure 2A – GSTm2 and GAPDH

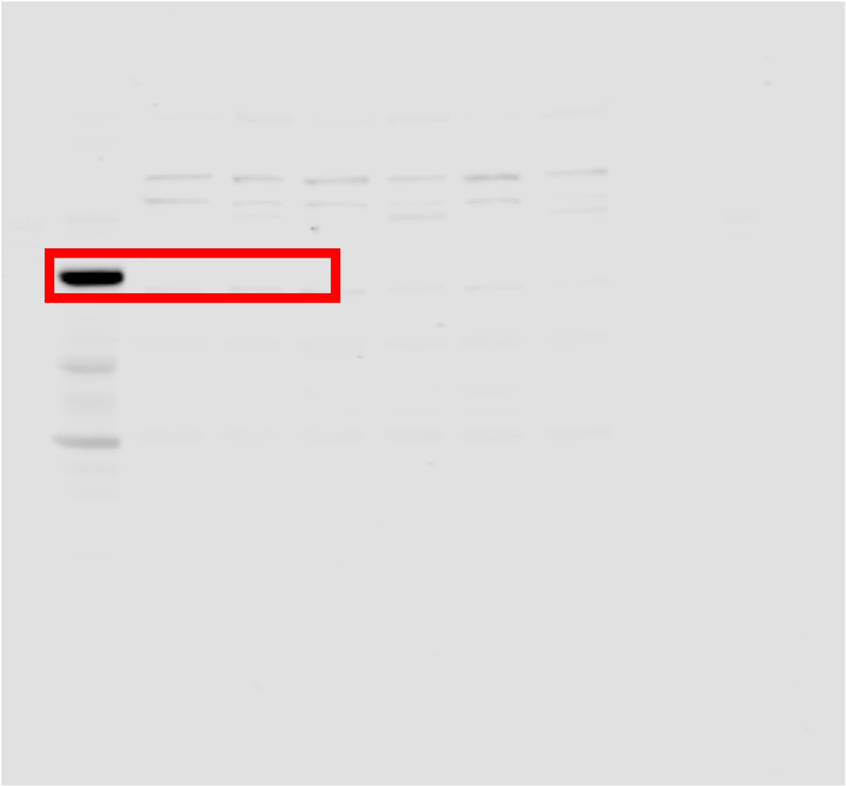

CBS

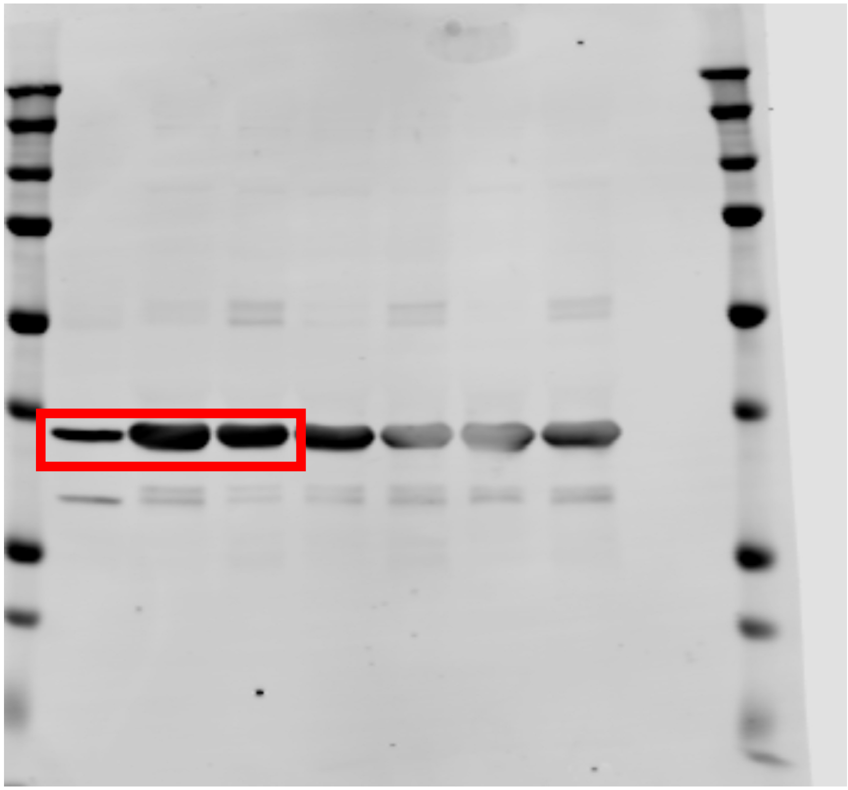

GAPDH (image used as representative for loading control)

Full unedited gel for Figure 3A – CBS and GAPDH

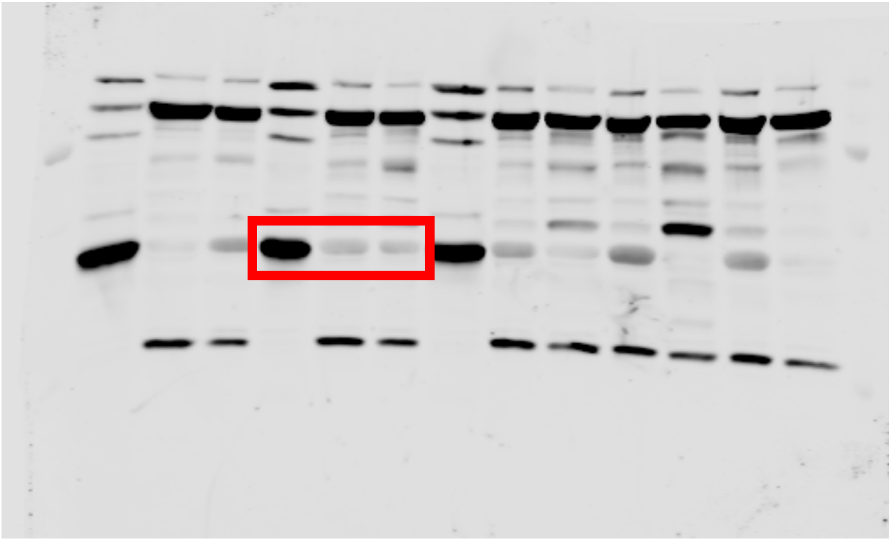

CSE

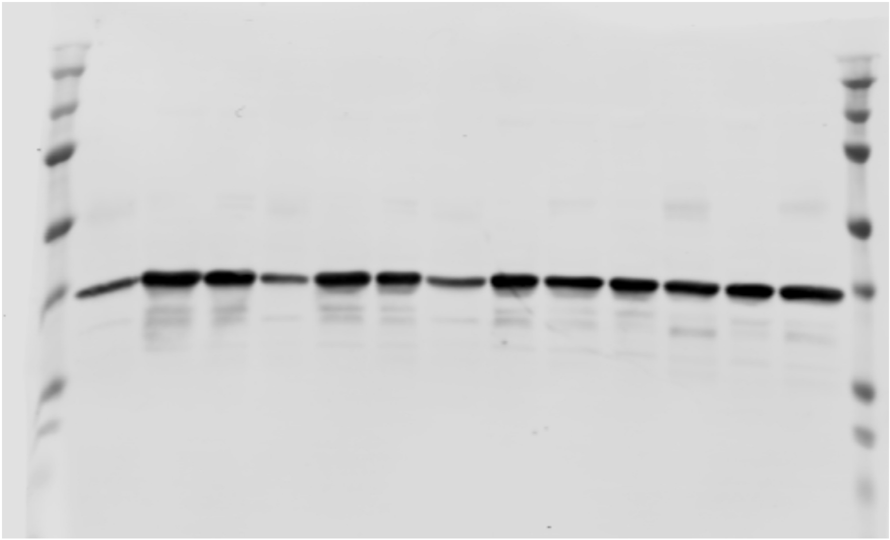

GAPDH (image not shown)

Full unedited gel for Figure 3A – CSE

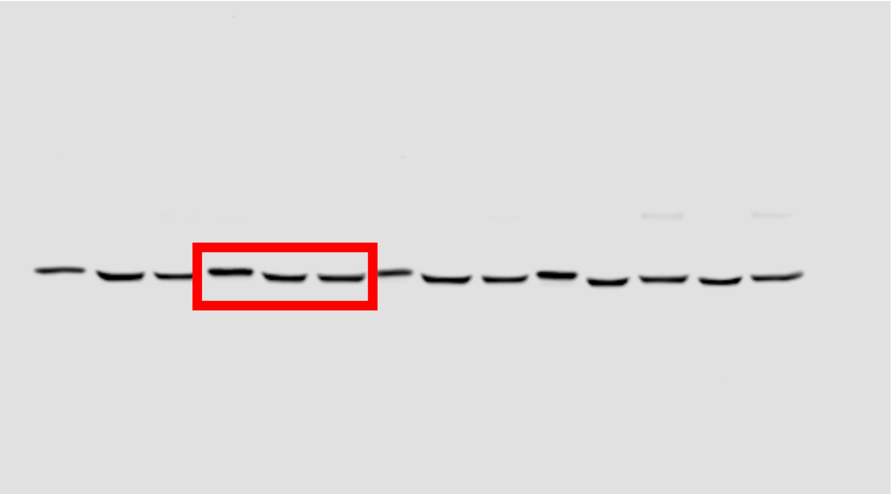

GOT1

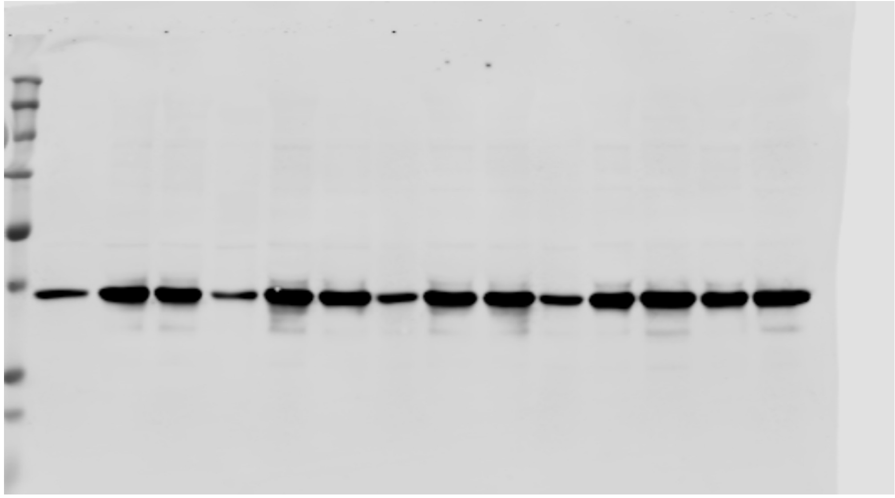

GAPDH (image not shown)

Full unedited gel for Figure 3A – GOT1 and GAPDH

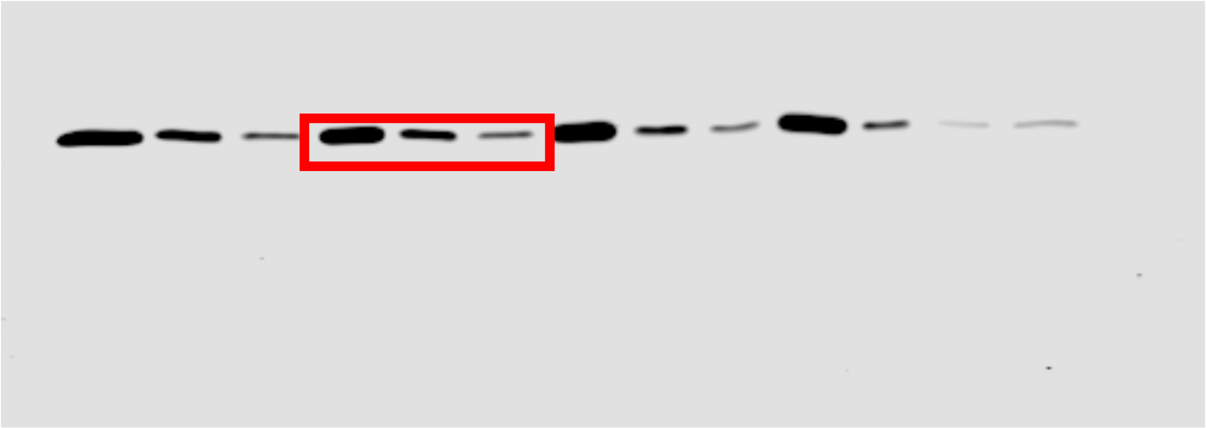

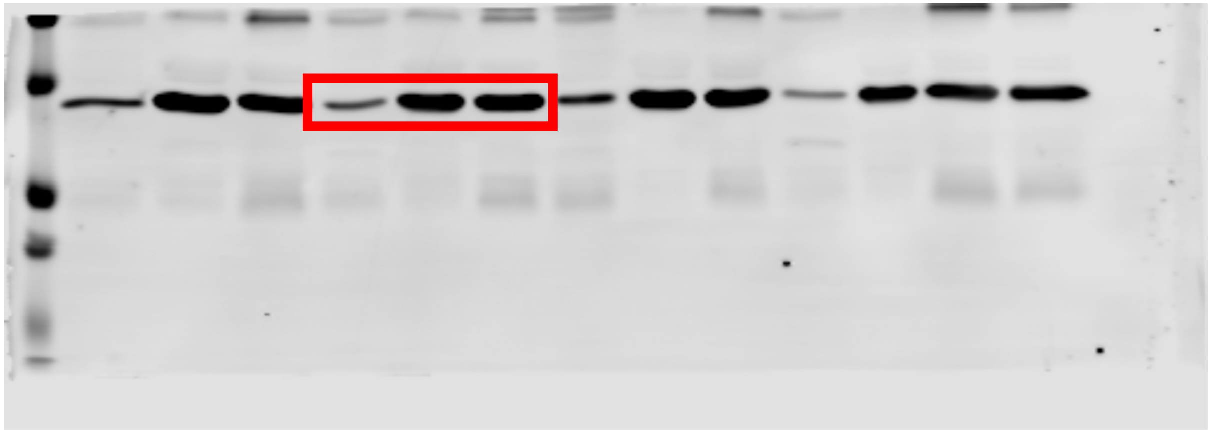

Full unedited gel for Figure 3A – MPST and GAPDH

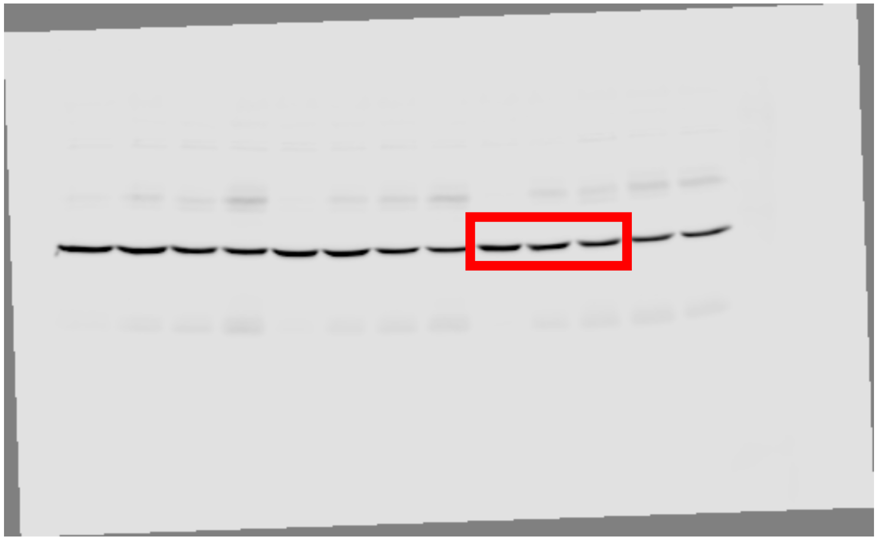

GOT1

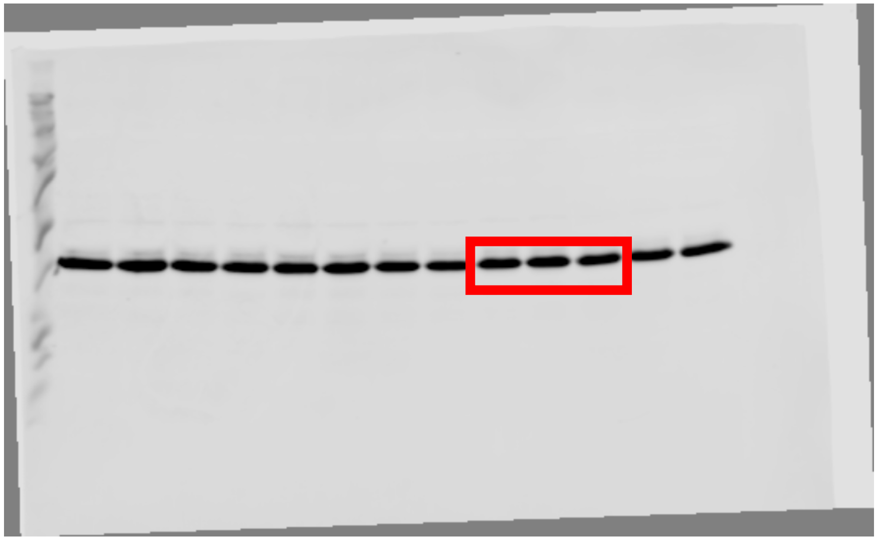

GAPDH (image used as representative for loading control)

Full unedited gel for Figure 3D – GOT1

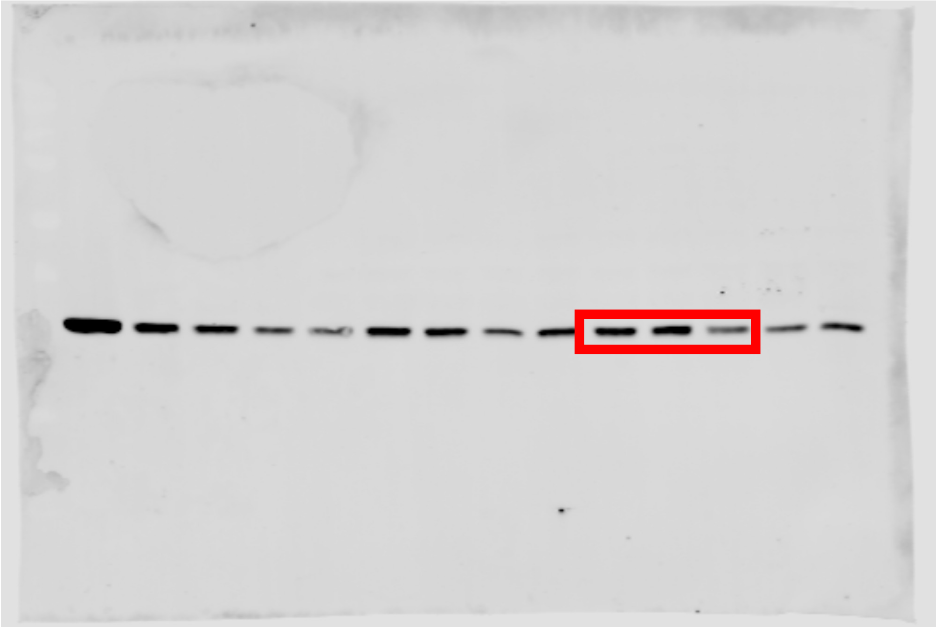

MPST

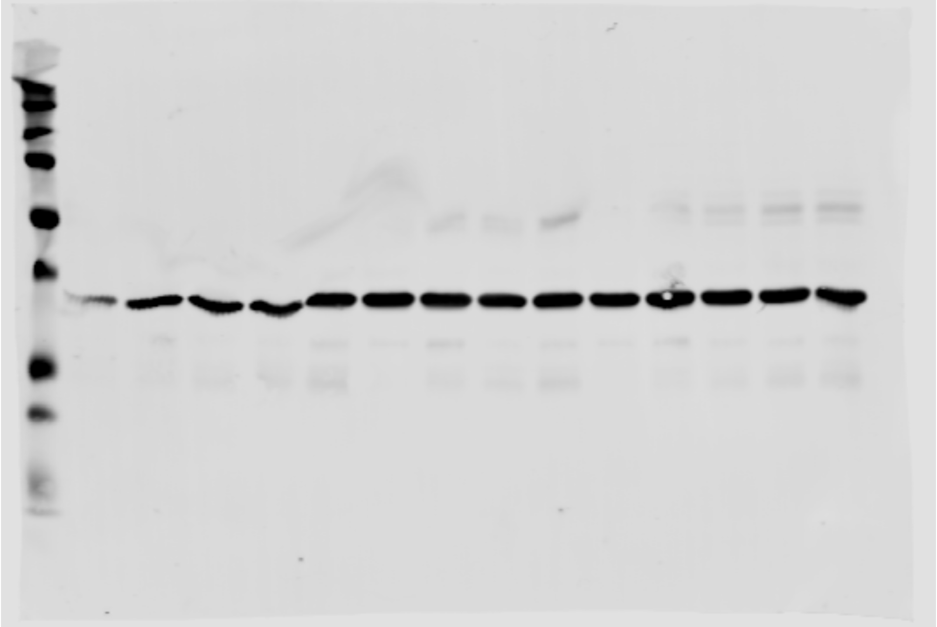

GAPDH (image not shown)

Full unedited gel for Figure 3D – MPST and GAPDH

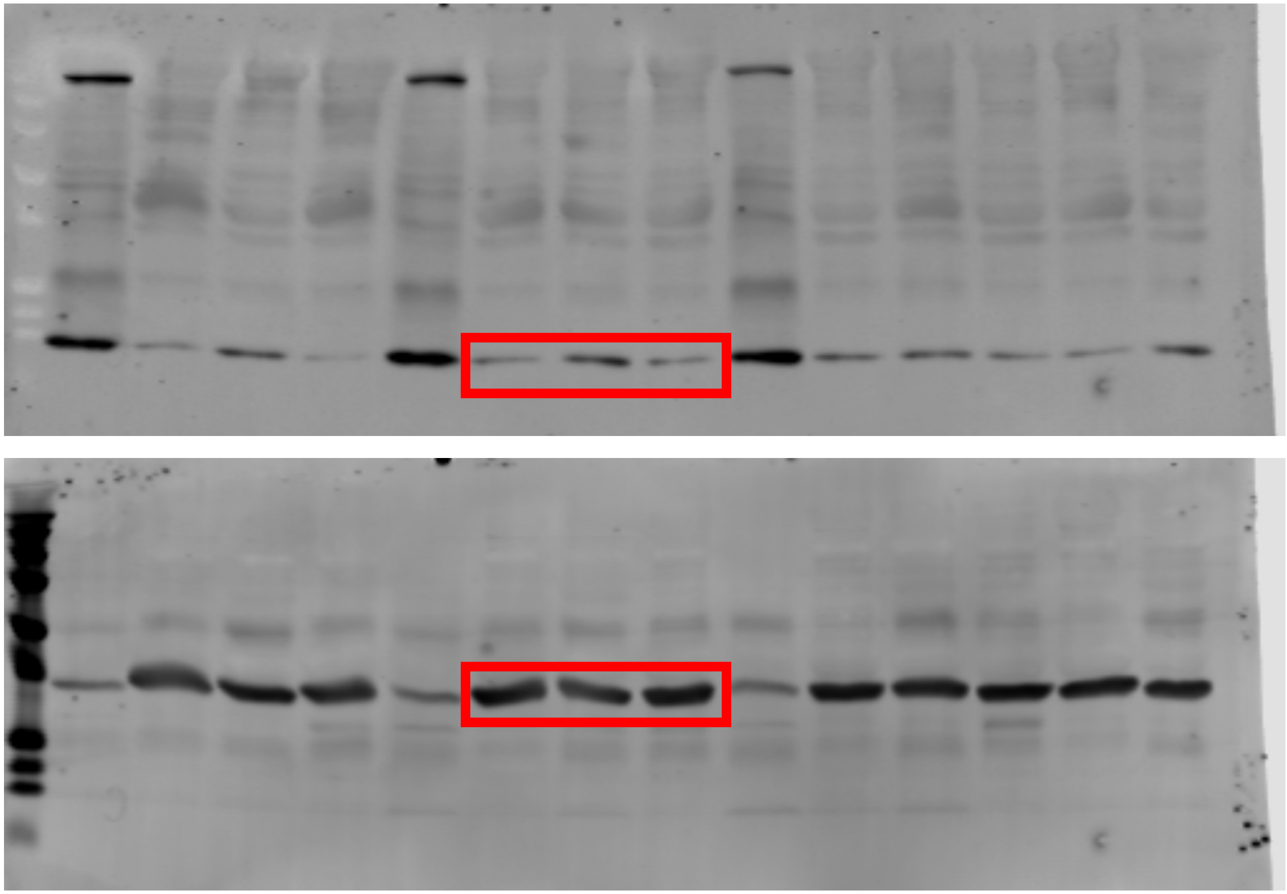

Full unedited gel for Figure 3E – TRX

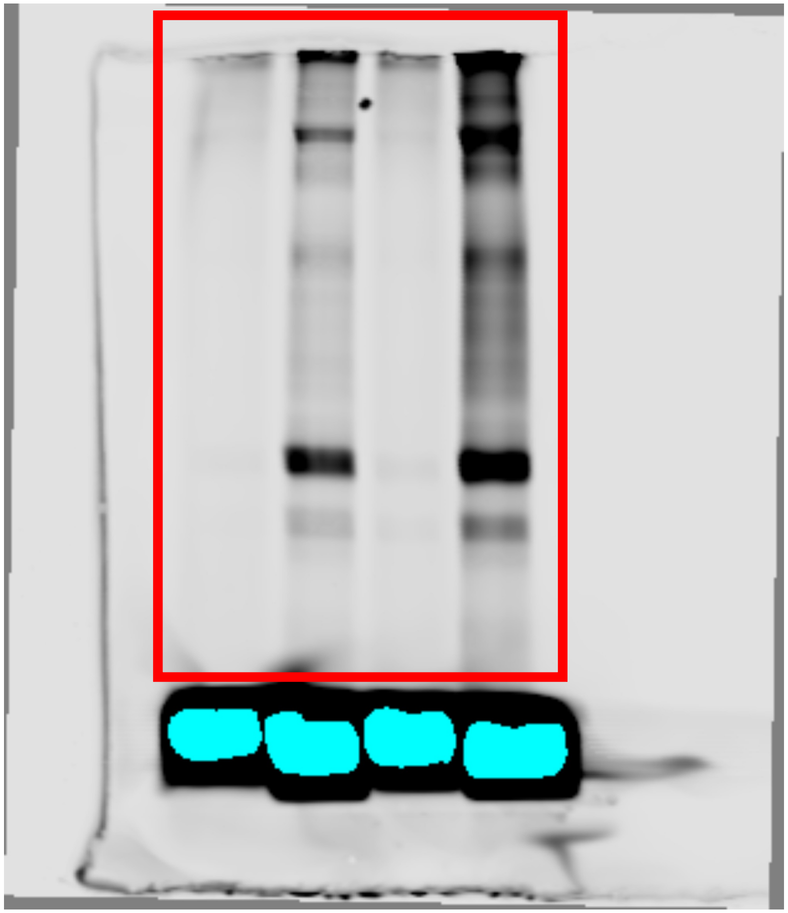

Full unedited gel for Figure 5A

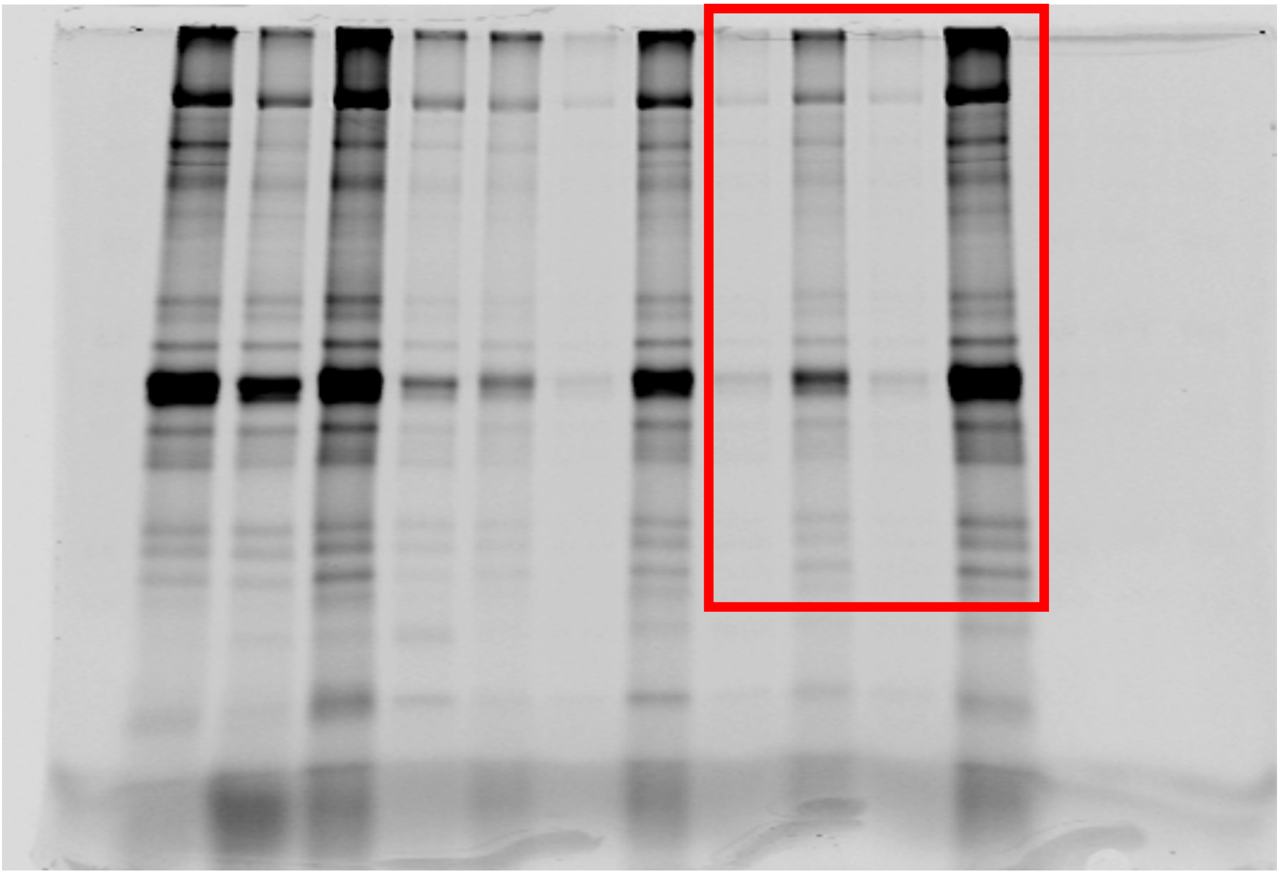

Full unedited gel for Figure 5D
